# *In-situ* proliferation contributes to the accumulation of myeloid cells in the spleen during progressive experimental visceral leishmaniasis

**DOI:** 10.1371/journal.pone.0242337

**Published:** 2020-11-12

**Authors:** E. Yaneth Osorio, Audrie A. Medina-Colorado, Bruno L. Travi, Peter C. Melby

**Affiliations:** 1 Department of Internal Medicine, University of Texas Medical Branch, Galveston, Texas, United States of America; 2 Department of Microbiology and Immunology, University of Texas Medical Branch, Galveston, Texas, United States of America; 3 Center for Tropical Diseases and Institute for Human Infection and Immunity, University of Texas Medical Branch, Galveston, Texas, United States of America; 4 Department of Pathology, University of Texas Medical Branch, Galveston, Texas, United States of America; Golestan University of Medical Sciences and Health Services, ISLAMIC REPUBLIC OF IRAN

## Abstract

Visceral leishmaniasis (VL) is characterized by expansion of myeloid cells in the liver and spleen, which leads to a severe splenomegaly associated with higher risk of mortality. This increased cellularity is thought to be a consequence of recruitment of cells to the viscera. We studied whether the local proliferation of splenic myeloid cells contributes to increased splenic cellularity. We found that a monocyte-like population of adherent splenic cells from *Leishmania donovani*-infected hamsters had enhanced replicative capacity *ex vivo* and *in vivo* (BrdU incorporation, p<0.0001). *In vitro* assays demonstrated that proliferation was more pronounced in the proinflammatory M1 environment and that intracellular infection prevented proliferation. Secondary analysis of the published splenic transcriptome in the hamster model of progressive VL revealed a gene expression signature that included division of tumoral cells (Z = 2.0), cell cycle progression (Z = 2.3), hematopoiesis (Z = 2.8), proliferation of stem cells (Z = 2.5) and overexpression of proto-oncogenes. Regulators of myeloid cell proliferation were predicted *in-silico* (CSF2, TLR4, IFNG, IL-6, IL-4, RTK signaling, and STAT3). The *in-silico* prediction was confirmed with chemical inhibitors of PI3K/AKT, MAPK and STAT3 which decreased splenic myeloid cell division *ex vivo*. Hamsters infected with *L*. *donovani* treated with a STAT3 inhibitor had reduced *in situ* splenic myeloid proliferation (p = 0.03) and parasite burden. We conclude that monocyte-like myeloid cells have increased STAT3-dependent proliferation in the spleen of hamsters with visceral leishmaniasis and that inhibition of STAT3 reduces myeloid cell proliferation and parasite burden.

## Introduction

Visceral leishmaniasis is caused by infection with the intracellular protozoan parasite *Leishmania*. *donovani* or *L*. *infantum*. Active, untreated VL is characterized by relentlessly progressive splenomegaly, pancytopenia, cachexia, and ultimately death. Diagnostic and post-mortem splenic biopsies of patients with VL demonstrate accumulation of myeloid cells and plasma cells in the white and red pulp of the spleen [1–4]. Massive splenomegaly is associated with increased risk of mortality in VL patients [4].

The mechanism(s) that drive accumulation of myeloid cells in the spleen in VL have not been clearly defined. It has been thought to be due to excessive recruitment of mature inflammatory myeloid cells from the bone marrow [5]. However, mechanisms such as extramedullary hematopoiesis and local cell proliferation may also contribute to this splenic myeloid cell accumulation.

Extramedullary hematopoiesis is a pathological process involving mobilization and differentiation of hematopoietic stem cells or progenitors outside the bone marrow [6]. It is a response to a hematopoietic crisis. Experimental studies show elevated numbers of myeloid progenitors in the spleen and bone marrow of BALB/c mice infected with *L*. *donovan*i [7] and *L*. major [8], as well as in hamsters infected with *L*. *donovani* [9]. Interestingly, the frequency of progenitors [7, 9] and progenitor cell cycling was more pronounced in the spleen than in the bone marrow of *L*. *donovani* infected mice [7].

Early studies hypothesized that *Leishmania* could infect and proliferate in newly generated immature safe targets that are less responsive to cytokine-mediated activation [8]. These experimental studies inferred that the local proliferation of myeloid cells have pathological consequences, but the actual role of local division of these cells in the pathogenesis of VL is uncertain.

Several pathological conditions, including infections, inflammation, cancer and obesity are associated with local proliferation and self-renewal capacity of myeloid cells [10–13]. Human peripheral blood (CD14)+ monocytes, differentiated with macrophage colony stimulating factor (M-CSF) or granulocyte-macrophage colony-stimulating factor (GM-CSF), proliferated after the *in vitro* infection with *L*. *major* [14]. Likewise, accumulation of hematopoietic growth factors, including GM-CSF, granulocyte colony-stimulating factor (G-CSF), and M-CSF was correlated with increased progenitors in the spleen of BALB/c mice infected with *L*. *donovani* [7].

The hamster model of VL is advantageous for study because it is a chronically progressive and ultimately fatal disease which mimics several aspects of the pathogenesis in human VL [15]. Our previous work demonstrated that the massive splenomegaly seen in the hamster model of progressive VL was accompanied by dramatic expansion of the myeloid cell population [16]. The current study demonstrates that this cellular expansion is fueled at least in part by *in situ* proliferation of myeloid cells. Splenic myeloid cell proliferation was driven and/or supported by factors produced by spleen cells early in the course of disease. Furthermore, the splenic environment in VL conditioned newly generated myeloid cells to be more supportive of parasite replication. Interrogation of the splenic myeloid cell transcriptome [17, 18] revealed a gene signature similar to proliferating tumor cells. This included dysregulated expression of proto-oncogenes and overactivation of PI3K/AKT, MAPK and STAT3 signaling pathways. Inhibition of STAT3 signaling reduced *in situ* proliferation, accumulation of splenic myeloid cells, and splenic parasite loads. These findings add new insight into the pathogenic generation of splenic myeloid cells and splenomegaly in visceral leishmaniasis.

## Materials and methods

### *In vitro* studies

#### Bone marrow derived macrophages (BMDM)

BMDM obtained from hamster femurs were differentiated with recombinant mouse M-CSF (20ng/ml) in culture medium (RPMI 1640 Glutamax, 10% heat inactivated fetal bovine serum-FBS, 55uM β-mercaptoethanol and 100U/mL Penicillin-100ug/mL Streptomycin) for 5–6 days. BMDM from adherent monolayers were infected 1:2 with *L*. *donovani* promastigotes (MHOM/SD/001S-2D) for 72h. DNA synthesis was determined after 2h of *ex vivo* pulse with 0.1mM bromodeoxyuridine (BrdU) at 37C 5% CO_2_. Proportion of cells that incorporated BrdU was evaluated by flow cytometry following the BrdU Flow kit instructions (BD Pharmingen). Isotype controls were used as threshold for analysis and uninfected samples as experimental controls. Mitosis was determined by expression of the nuclear antigen ki-67 using the rabbit anti-ki-67 antibody (0.8μg/10^6^ cells AB15580, Abcam, overnight, 4°C). Ki-67 antibody was directly labeled with rabbit Zenon Alexa Fluor 647 (Z25308, ThermoFisher Sci.) or with 0.1μg goat anti-Rabbit IgG (H&L) allophycocyanin preabsorbed conjugate (AB130805, Abcam, 30 min, 4C). Number of cells in mitosis was calculated by the percentage of cells expressing ki-67 x number of cells /100. Polarized M1 and M2 BMDM were obtained *in vitro* with rat GM-CSF (20ng/mL) or mouse M-CSF (20ng/mL) after 5 days [19]. Cells were stimulated with mouse insulin like growth factor 1 (IGF-1) (100ng/mL), basic fibroblast growth factor 2 (FGF-2) (20ng/mL), hamster interleukin 4 (IL-4) (5U/mL of recombinant IL-4 supernatant) [20]. The proportion of cells in division was determined by microscopy or flow cytometry as above.

#### Proliferative *L*. *donovani* parasites

Promastigotes were stained with CellTrace Far Red Cell Proliferation Kit (ThermoFisher Sci.) and then added 1:1 to M1 or M2-polarized BMDM monolayers. Extracellular parasites were washed out at 1h and 48h p.i. (post-infection) for at least 4 washes with warm PBS until no evident extracellular parasites were observed. The presence of intracellular parasites was evaluated by flow cytometry. Proliferative parasites were assessed by the diluted fluorescence of the probe.

#### Interference RNA (RNAi)

RNAi was performed with 6nM of either signal transducer and activator of transcription 6 (STAT6) short interfering RNA (siRNA) or negative control oligonucleotides plus Lipofectamine® RNAiMAX (ThermoFisher Sci.) [21]. After 72h of RNAi, BMDM were stimulated with *Leishmania* promastigotes and GM-CSF.

### Chemical inhibitors

Chemical inhibitors were used at concentrations causing less than 90% toxicity to either BMDM or splenic cells as follows: 1.5μM PI3-kinase inhibitor (LY29402), 10μM MEK1/2 kinase inhibitor (PD98059), 0.2μM AKT inhibitor (MK-2206); 25μM signal transducer and activator of transcription 3 (STAT3) inhibitor (S31-201) and evaluated with reference to cells treated with each final concentration of vehicle DMSO.

### *Ex vivo* studies

Spleens from infected hamsters (28 days p.i.) were treated with 2mg/mL collagenase and 20μg/mL DNase for 20min. Splenic cells were released through a 100μM mesh, counted, washed and allowed to adhere for 1h at 37C 5% CO_2_ in RPMI 5% FBS. After discarding the non-adherent fraction, the adherent monolayer was washed at least 4 times with warm PBS. The adherent cells were detached with a cell scraper, washed and stained with FITC antibodies to exclude any remaining CD3 T cells, MHCII+ dendritic cells, and B cells [16]. Expression of ki-67 and BrdU was compared with similar cells from uninfected hamsters as above. Adherent splenic cells were identified as monocyte/macrophage like-myeloid cells according to the morphology, CD68 positivity and gene expression profile [17]. The effect of chemicals inhibitors on proliferation was evaluated after 72h of *ex vivo* treatment with the chemical inhibitors as above.

### *In vivo* studies

#### Hamster infection

The Institutional Animal Care and Use Committee (IACUC) representing the University of Texas Medical Branch (UTMB) approved the study protocol. Golden Syrian female hamsters were maintained in clear cages with 3–4 hamsters per cage in BSL2 facilities in agreement to IACUC approved protocol. Hamsters were anesthetized with isoflurane vaporizer and infected IC with 10^6^ peanut agglutinin (PNA) purified metacyclic *L*. *donovani* promastigotes in 50μL PBS.

#### Pulse chase with BrdU

The *in-situ* proliferation was determined by the pulse chase method with BrdU. In brief, BrdU (Sigma, 200mg/kg/1mL PBS) was injected by the intraperitoneal route (i.p.) to infected hamsters. After 30 min of BrdU injection, hamsters were euthanized with CO_2_. After spleen removal, splenic adherent cells were isolated and counted as above. Adherent cells were stained with the BrdU flow kit and analyzed by flow cytometry.

#### Treatments with STAT3 inhibitor

Signal transducer and activator of transcription 3 (STAT3) inhibitor Cucurbitacin I (Sigma) or Stattic (Selleckchem) was given to *L*. *donovani-*infected hamsters (2–3 weeks p.i.) at a dose of 0.5mg/kg and 10mg/Kg respectively. Dose was given by the intraperitoneal route (i.p.) in 100–200μl volume of 10% DMSO: PBS, every other day, for two weeks. At the end of the treatment, animals were euthanized with CO_2_ inhalation. The spleen was collected to determine the number of cells (microscopy). The splenic parasite burden was determined by expression of 18S Leishmania RNA by RT-qPCR and the standard curve method [17].

### Colony formation assay

Colony formation assays (CFA) were performed with 25,000 cells of adherent splenic cells or BMDM seeded in petri dishes containing 3mL methylcellulose soft agar medium (optimized for enumeration of myeloid progenitor cells, without erythropoietin, to exclude the differentiation of erythrocytes) (HSC004, R&D). Some cultures were supplemented with 10% of conditioned medium (CM) obtained from supernatants from either adherent or total erythrocyte-free splenic cells from uninfected or infected hamsters. In brief, cells were cultured at a density of 2.5-5x10^6^/mL for 24h and then supernatants were collected and 10X concentrated through a 30,000 Kd protein concentrator (Millipore). After 12 days of culture in CFA plates, the number of colonies per field was estimated on the inverted microscope. Colonies were recovered after spinning the cells collected from the soft agar by dilution in 10 volumes of PBS. The total number of cells was determined by luminometry (Cell Titer Glo, Promega).

### Transcriptional profile by RNAseq

Genes involved in cell proliferation were identified in the annotated splenic monocyte/macrophage transcriptome of Golden hamsters *(Mesocricetus auratus)* infected with *L*. *donovani* [17]. Fold change of RNAseq reads was calculated with reference to uninfected samples with a cut-off of two-fold change and a false discovered ratio (FDR) <0.05. Functions significantly affected by differentially expressed genes were identified with the Ingenuity Pathway Analysis (IPA) software (Z score >2.0). Gene subsets implicated in significantly affected functions were extracted to study mechanisms of dysregulation such as cell proliferation of cancer cells (278 genes) and growth of tumors (106 genes). Subsets of Proto-oncogenes (72 genes) and tumor suppressor genes (44 genes) which regulate the cell cycle were identified in the transcriptome for IPA analysis.

### Statistical methods

Data were analyzed using InStat (v. 3.0) and Prism 5.0 (Graphpad, La Jolla CA) Software. Statistical tests were applied according to software recommendations. All experiments were repeated at least twice with 4–8 replicates per group, per experiment. Statistical significance is defined at each figure.

### Gene expression by qPCR

Primers were designed with the program primer-Blast (NCBI) using the hamster RNA sequence (National Center for Biotechnology, NCBI) (c-KIT for: ATCCTGACGTCTGAGAGGCT; c-KIT rev: TGGGGCTGGATTTGCTCTTT; IGF-1 for: AAATCAGCCCGCTCTATCCG; IGF-1 rev: TCAAAGGATCCTGCTGTCGC; Cyclin A for: ATGCCCTGGCCTTTAATGCT; Cyclin A rev: GGATGGCCCGCATACTGTTA; KLF4 for: GCCCAACTACCCTCCTTTCC; KLF4 rev: GGCTTCTCACCTGTGTGAGTT; OCT3/4 for: GGACACCTGGCTTCAGACTT; OCT3/4 rev: AACCTGAGGTCCGCAGTATG). RNA from splenic monocyte-like myeloid cells or total spleen of uninfected and infected hamsters was isolated with the RNeasy kit (Qiagen), then treated with DNase I (Turbo DNase, Ambion) and reverse transcribed (High capacity Reverse transcription kit, Thermofisher Sci.). Genes amplified by qPCR were detected with SYBR (Bio-Rad) to estimate the fold change of gene expression using the delta-delta-CT method with host 18S as reference gene.

## Results

### Splenic myeloid cells proliferate in VL

**Fig 1:**
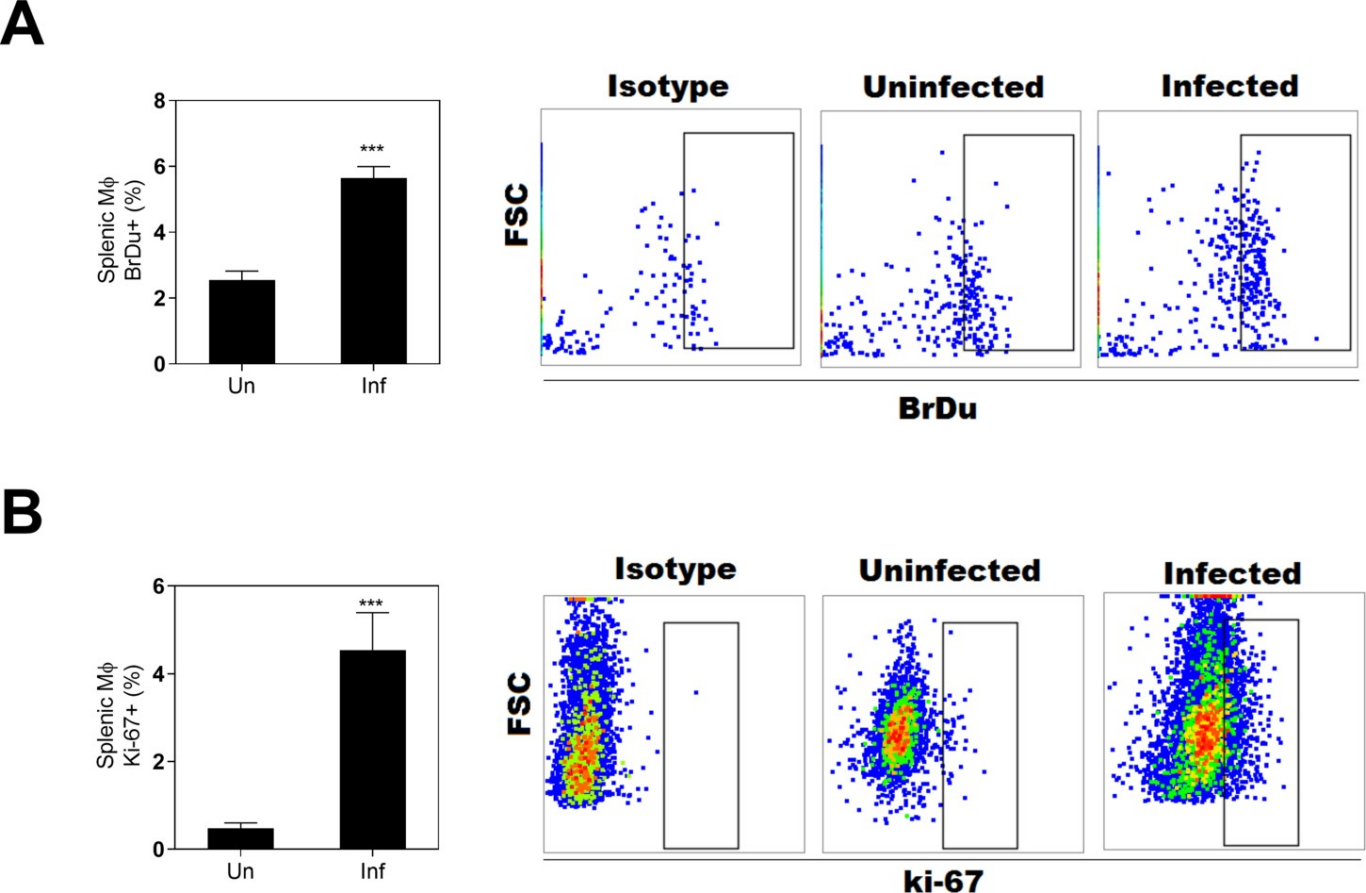
Splenic myeloid cells from hamsters infected with *L*. *donovani* are proliferative. **A,** Proportion of splenic myeloid cells BrdU+ (DNA synthesis) in uninfected (Un) or infected hamsters (Inf) at 28 d p.i., (*** p<0.0001, Unpaired T-test) and representative flow cytometry dot-plot showing the BrdU+ cells. **B,** Proportion of splenic myeloid cells in mitosis (Ki-67+), at 28d p.i., (*** p<0.0001, Mann-Whitney Test) and representative flow cytometry dot-plot showing the proportion of ki-67+ cells.

We investigated an established animal model that mimics the progressive nature of human VL to determine whether in situ myeloid cell proliferation contributes their accumulation in the spleen after the infection. We found splenic myeloid cells from hamsters infected with *L*. *donovani* had heightened proliferation compared with uninfected controls, as determined by *in vivo* BrdU incorporation into DNA and expression of the cellular mitosis marker ki-67 (**Fig 1A and 1B**). These approaches indicated that about 4–6% of these splenic myeloid cells were proliferative. Our previous studies showed that >90% of these adherent splenic cells are positive for the monocyte-macrophage marker CD68 by immunohistochemistry and express monocyte/macrophage lineage markers MMP12 and CSFR1 [17]. Since antibodies are not available to clearly define these cells, we refer to them generically as splenic myeloid cells.

**Fig 2:**
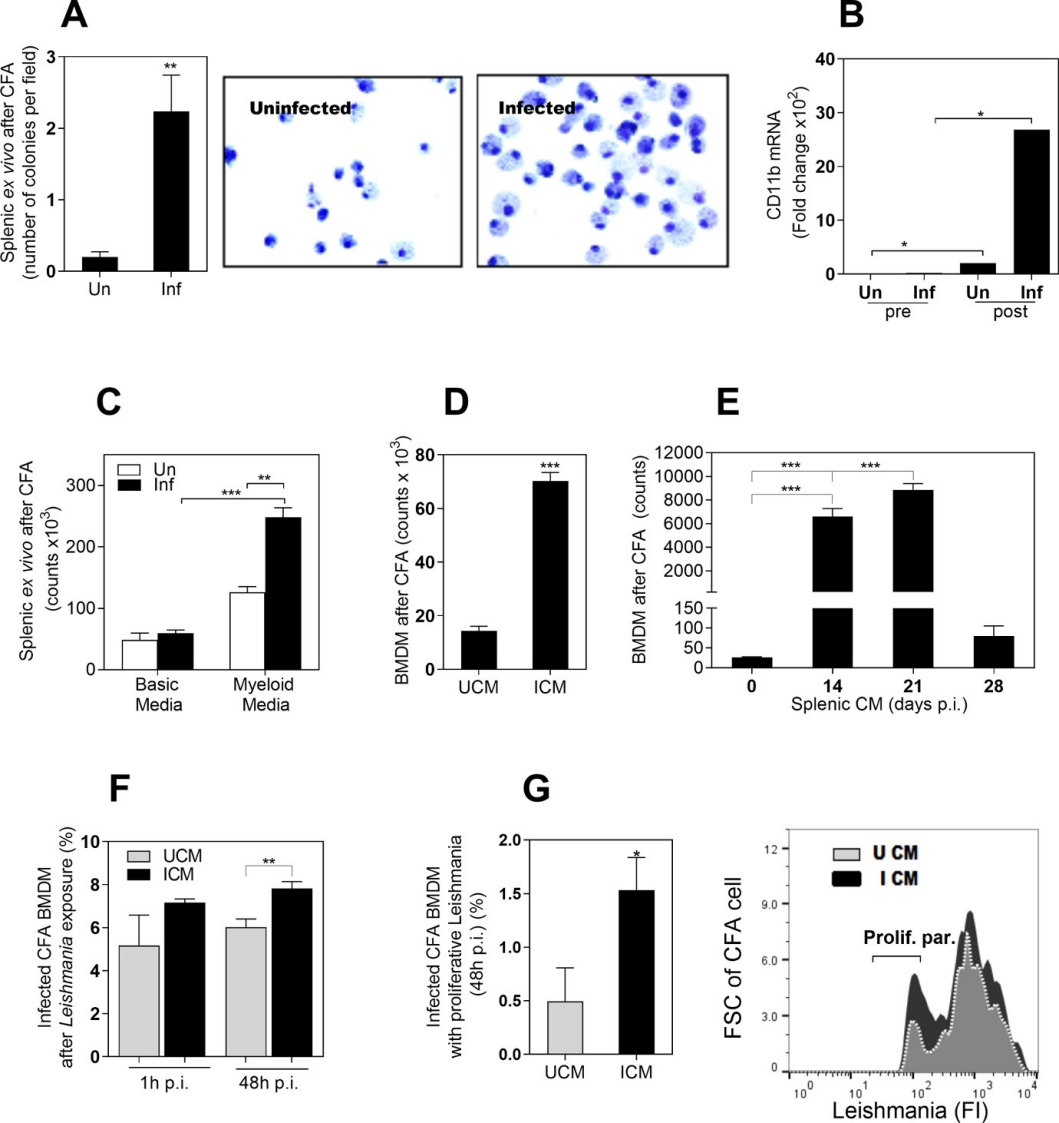
The infected environment in VL induces the proliferation of myeloid cells. **A,** Number of colonies in a colony formation assay (CFA) using splenic myeloid cells from uninfected hamsters (Un) or from hamsters infected with *L*. *donovani* (Inf). Colonies were counted on the inverted microscope after 12 days of incubation (**p<0.008, Mann-Whitney Test); Right, representative Giemsa staining of cells at day 12 of CFA, showing some bi-nucleated cells; **B,** Expression of the pan-myeloid CD11b marker before culture (pre) and at 12 days of CFA (post), determined by qPCR (*p<0.05, Kruskal-Wallis Test); **C,** Number of cells at 12 days of CFA with splenic myeloid cells exposed to a basic medium (without myeloid growth factors) or complete medium (with myeloid growth factors). The number of cells was determined by Cell Titer Glo (**p<0.01; ***p<0.0001, Kruskal-Wallis Test); **D,** Number of bone marrow derived cells (BMDM) at 12 days of exposure to a CFA with CM from spleen of uninfected (UCM) or infected hamsters (ICM) (***p<0.0001, Unpaired T test); **E,** Number of BMDM at 12 days of CFA exposed to splenic CM obtained from spleens of uninfected hamsters (time 0) or from hamsters infected with *L*. *donovani* at the indicated times post-infection (14–28 days p.i.) ***p<0.001, Tukey-Kramer Multiple Comparisons Test); **F,** Percentage of infected CFA cells after in vitro exposure to *L*. *donovani* promastigotes (1h or 48h of in vitro infection). CFA cells obtained after 12 days of BMDM exposed to UCM or ICM as determined by flow cytometry (**p = 0.03, Unpaired t test); **G,** Percentage of cells from CFA with proliferative parasites after 48h of in vitro infection with *L*. *donovani* promastigotes. Parasites were prelabeled with a fluorescent proliferation tracer before in vitro infection (*p< 0.004, Unpaired T test). Right, representative histogram showing fluorescence intensity of proliferative parasites (with diluted fluorescence) compared with parental parasites.

We next tested the capacity of splenic myeloid cells to proliferate in a myeloid cell colony formation assay (CFA). We found that splenic myeloid cells isolated from infected hamsters produced more colonies than those isolated from uninfected hamsters after 12 days of *ex vivo* culture (**Fig 2A**). Daughter cells from those colonies were identified as monocyte-like myeloid cells by morphology (**Fig 2A**) and by the expression of the pan-myeloid marker CD11b (**Fig 2B**). Commercial CFA medium, containing myeloid specific growth factors (HSC004, R&D), supported myeloid proliferation compared with base medium (HSC002, R&D) (**Fig 2C**).

We also established a model of proliferation with bone marrow derived macrophages (BMDM) and studied factors that affect the proliferation of these cells. We first observed that BMDM proliferated after exposure to viable *L*. *donovani*, determined by increase in cell number (**S1A Fig in S1 File**), incorporation of BrdU (**S1B Fig in S1 File**) and expression of the mitosis marker ki-67 (**S1C Fig in S1 File**). To determine if the environment of the infected spleen promoted the proliferation of BMDMs, we exposed these cells in a colony forming assay to CM from cultures of spleen cells derived from infected or uninfected animals. Exposure to CM from infected spleen cells resulted in significantly greater number of cells than exposure to CM from uninfected spleen cells (**Fig 2D**). To evaluate whether the evolution of the infection affected the intensity of cell division, we exposed BMDM from uninfected hamsters to CM obtained from spleen cells collected from infected hamsters at different times after infection. We found that splenic CM from animals at 14- and 21-days post-infection induced the highest *in vitro* proliferation of BMDM compared with uninfected control splenic CM (~90-fold increase; **Fig 2E**). Surprisingly, spleen cell CM from 28-day infected hamsters induced proliferation in BMDM at a level just ~2-fold over CM from uninfected hamsters. This occurred even though the increase in spleen size continues beyond 56 days post-infection [15].

### Cells produced in the infected environment are more permissive to *L*. *donovani* replication

To know if cells generated in the infected environment were more permissive to *Leishmania*, we cultured BMDM with CM from either uninfected or infected hamster spleen cells. After 12 days of culture, we exposed those daughter BMDM to *L*. *donovani* pre-stained with a fluorescent cell-proliferation tracer. We found similar rates of infection at 1h post-infection (entry of parasite to the cell) (**Fig 2F**). However, after 48h of infection a higher percentage of cells generated in the infected splenic CM contained intracellular *Leishmania* (**Fig 2F**). Analysis of proliferative parasites, defined by diluted fluorescence of the cell tracer, showed a higher proportion of proliferating parasites in BMDMs generated after exposure to splenic CM from infected animals than in those exposed to splenic CM from uninfected animals (**Fig 2G**). This suggests that the splenic environment in VL conditions newly generated myeloid cells more supportive of parasite replication.

### Polarization affects the proliferative capacity

**Fig 3:**
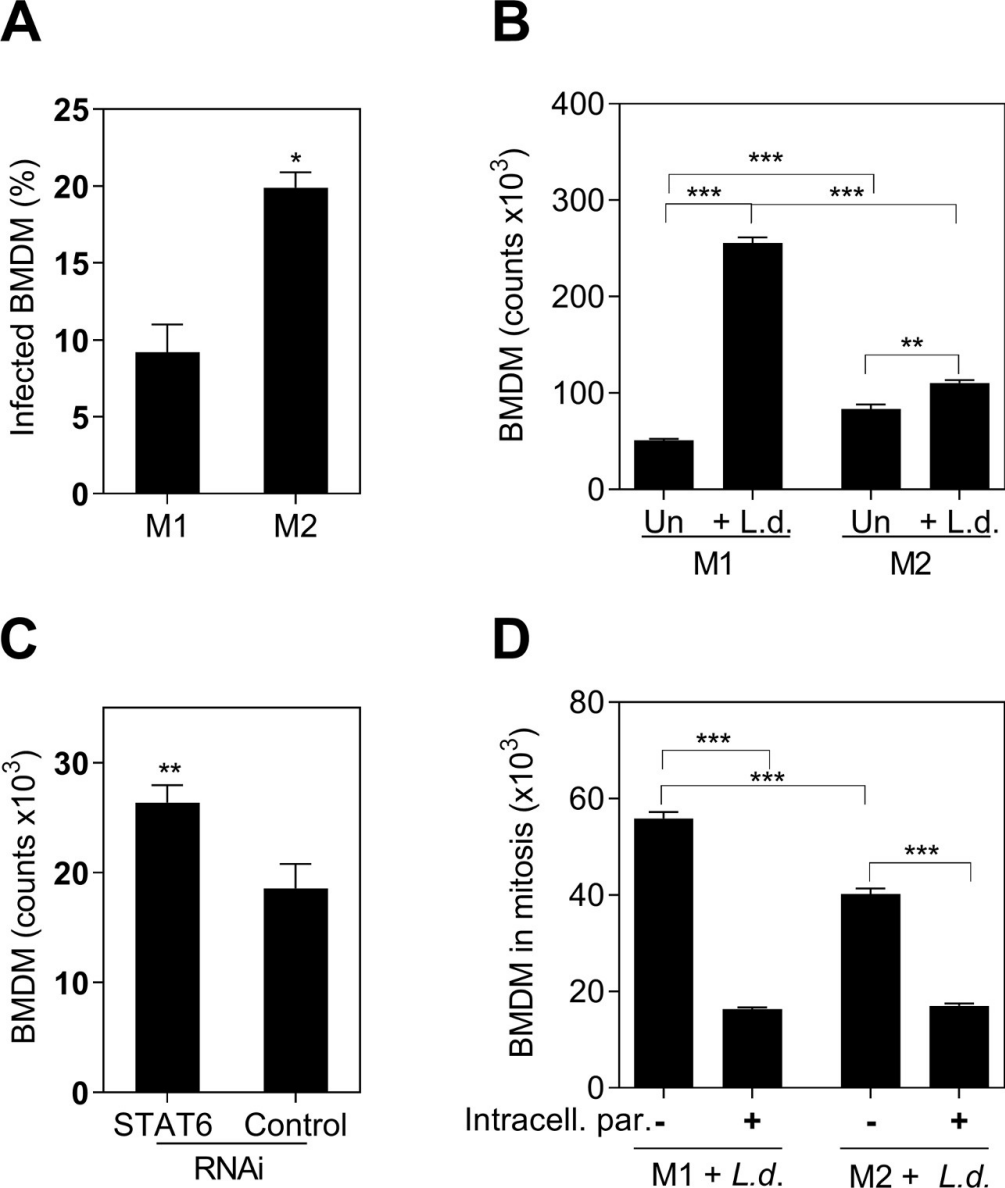
The proliferative capacity of BMDM was limited by intracellular *L*.*donovani* parasites and M2-polarization. **A,** Proportion of infected M1 or M2 BMDM after 48 hours of exposure to *L*. *donovani* promastigotes (*p = 0.03, Unpaired t test, representative of 3 experiments); **B,** Absolute number of M1 or M2 BMDM stimulated with *L*. *donovani* promastigotes (+L.d.) or without parasite stimulation (Un). Number of cells counted by luminometry (*** p<0.001, **p<0.01, Tukey-Kramer Multiple Comparisons Test, representative of 3 different experiments); **C,** Number of BMDM after STAT6 silencing (STAT6 RNAi) compared with control silenced cells (control RNAi), 48h after exposure to *L*. *donovani* parasites (** p = 0.0079, Mann-Whitney Test). Data is the number of stimulated cells minus non-stimulated cells; **D,** Number of M1 or M2-polarized BMDM Ki-67+ (mitosis) containing intracellular parasites (+) or remaining free of intracellular parasites (-), after 48h of exposure to fluorescent-*L*. *donovani* (Number of cells in mitosis = percentage ki-67+ cells by flow cytometry x number of cells by luminometry/ 100) (*** p<0.0001, Tukey-Kramer Multiple Comparisons Test, representative of 3 different experiments).

To evaluate whether the polarization status of the myeloid cells affected their proliferation, we polarized BMDM toward M1 and M2 phenotypes according to standard methods [22]. We confirmed that in our system, M2-polarized BMDMs had greater susceptibility *to L*. *donovani* infection than did the M1-polarized BMDMs (**Fig 3A**). Both M1 and M2 BMDMs increased in number after exposure to *L*. *donovani* for 48 hrs. However, the number of M1-polarized BMDM was significantly greater than M2-polarized BMDM (**Fig 3B**). To further test that M1 polarization favored proliferation, we silenced the regulator of M2 activation STAT6, using RNA interference (RNAi) [21]. We found that STAT6 RNAi led to enhanced cell proliferation compared with control RNAi (**Fig 3C**). Collectively, these data support the inverse relationship between M2 polarization and proliferation.

To determine whether intracellular parasites affect the proliferative capacity of BMDM, we exposed M1 and M2-polarized BMDM to pre-labeled *L*. *donovani* parasites and identified mitotic Ki-67+ BMDM containing intracellular parasites. We found that most cells in mitosis, whether they were M1- or M2-polarized, were free of intracellular parasites (**Fig 3D**) indicating that intracellular *Leishmania* amastigotes impaired cell proliferation.

### Genes associated with myeloid cell proliferation had a signature consistent with tumor cells

With the evidence that splenic myeloid cells expand *in situ*, we analyzed the published transcriptome of these cells [17] using Ingenuity Pathway Analysis (IPA) software. The software predicted activation of functions according to the number and pattern of differentially expressed genes associated with that function (activation Z score >2.0). Functions associated with replication of tumor cell lines, hematopoiesis of phagocytes, cell differentiation of phagocytes and quantity of hematopoietic progenitors were predicted to be activated in the splenic myeloid cell transcriptome (Z score >2.0) (**Table 1**). To identify potential mechanisms that lead to myeloid proliferation, we overlaid genes differentially expressed (>2.0 fold change infected vs. uninfected splenic myeloid) with the set of 278 genes expressed during cell proliferation of tumor cell lines in the IPA data base (Z = 2.1, p = 2.45E-27) (**Table 1**).

**Table 1:** Functions associated with phagocytes and cell proliferation identified in the transcriptome of splenic myeloid cells from hamsters with VL.

Functions	p-Value	Activation State	Activation z-score	# Molecules
Cell proliferation of tumor cell lines	2.45E-271	Increased	2.1	278
Hematopoiesis of mononuclear leukocytes	2.58E-41	Increased	2.9	80
Differentiation of mononuclear leukocytes	4.15E-42	Increased	2.8	81
Differentiation of phagocytes	1.52E-21	Increased	2.0	35
Hematopoiesis of phagocytes	4.56E-21	Increased	2.0	30
Infiltration by mononuclear leukocytes	7.52E-21	Increased	2.9	29
Chemotaxis of phagocytes	9.77E-21	Increased	2.7	36
Quantity of hematopoietic progenitor cells	2.86E-36	Increased	2.1	60

p-Value, Fisher’s Exact test to reflect the likelihood that the association between molecules and a given process is due to random chance. Activation state and Z score predicted with IPA.

**Fig 4:**
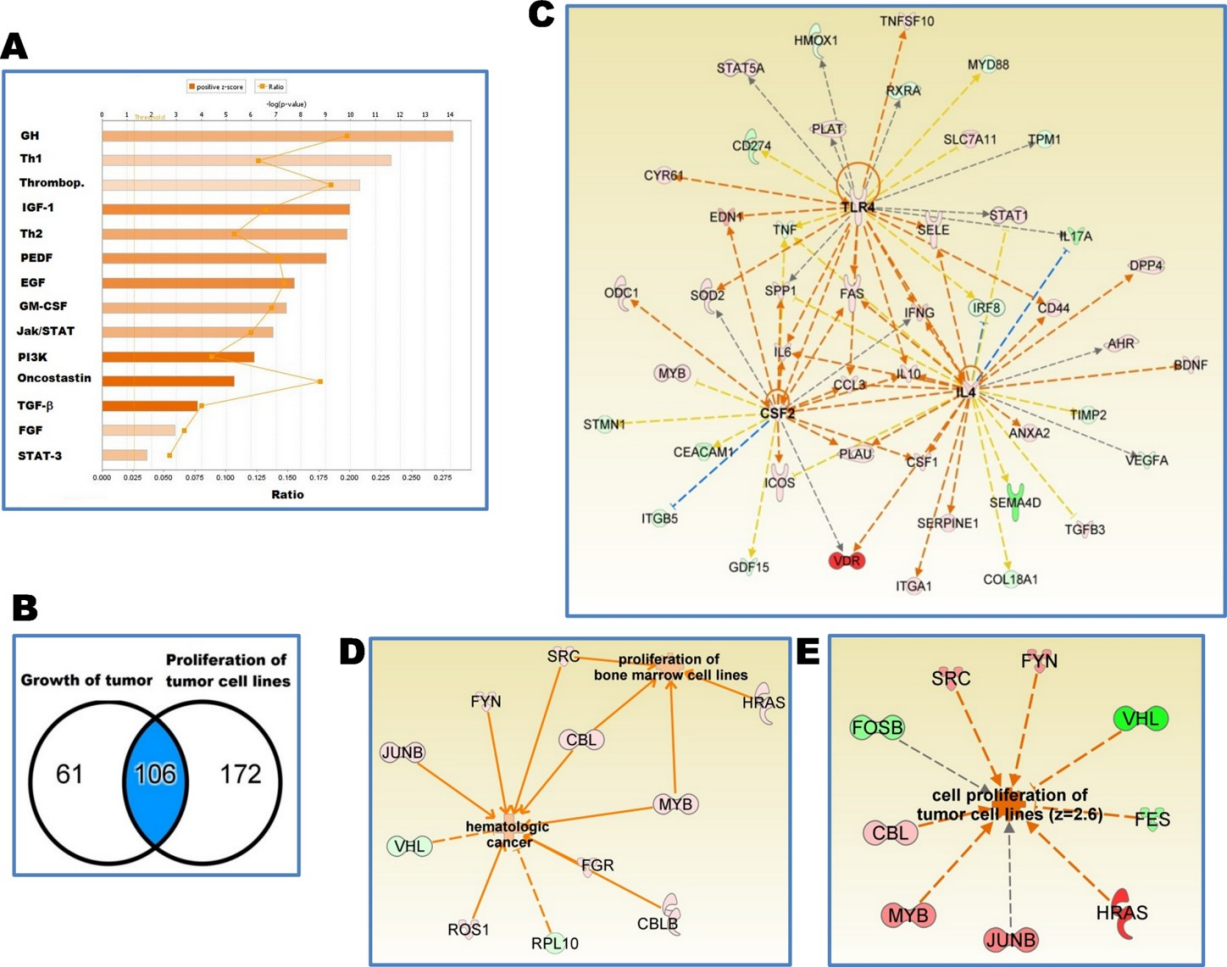
Transcriptome of splenic myeloid cells from hamsters with VL show proliferative pathways characteristic of cancer diseases. **A**, Signaling pathways associated with cell proliferation of tumor cell lines in a set of 278 differentially expressed genes identified in the transcriptome of splenic myeloid cells of hamsters with VL; **B**, Venn diagram representing a set of 106 differentially expressed genes associated with functions of proliferation of tumor cell lines and growth of tumor; **C**, Predicted upstream regulators of those 106 differentially expressed genes: Interleukin-4 (IL-4, Z = 2.32), Granulocyte-macrophage colony-stimulating factor = Colony stimulating factor 2 (CSF2, Z = 2.0) and Toll-like receptor 4 (TLR4, Z = 2.32); Orange arrows: lead to activation; blue arrows: lead to inhibition; yellow arrows: conflicting finding; grey arrows undetermined; pink to red gene: increased expression; green gene: decreased expression; **D**,**E**, Differentially expressed proto-oncogenes and tumor suppressor genes that lead to cell division of tumoral cells. Relationships and functions predicted with the IPA software (Z score > 2.0). Differential gene expression found in the hamster splenic myeloid transcriptome [17] with a FDR<0.05 and fold change 2.0 with reference to uninfected.

A total of 141 of those 278 genes (51%, Z = 2.05, p = 2.45E-27) had a direction consistent with dysregulated cell proliferation typical of tumor cell lines (**S1 Table in S1 File**). Analysis of those genes indicated that the growth factor signaling pathways represented by growth hormone (GH), IGF-1, epidermal growth factor (EGF), and FGF, pathways were activated (**Fig 4A**). Th1 signaling, phosphoinositide 3-kinase (PI3K) and STAT3 signaling pathways were also activated.

To identify global upstream regulators of cell proliferation we narrowed down the analysis to a subset of 106 common genes associated with tumor growth and proliferation of tumor cell lines (**Fig 4B**). Proto-oncogenes HRAS and JUNB were predicted upstream regulators of differentially expressed genes associated to splenic myeloid cells proliferation (**Table 2**). Additionally, GM-CSF (CSF2), IL-4 and toll like receptor 4 (TLR4) were central to gene expression networks of growth and proliferation (**Fig 4C** and **Table 2** and **S2 Table in S1 File**). The same analysis show 28 of 45 genes differentially expressed in a direction consistent with proliferation of hematopoietic cells (62%, Z = 2.83, p = 4.71E-32) (**S3 Table in S1 File**), 32 of 59 genes differentially expressed in a direction consistent with cell cycle progression (54%, Z = 2.34, p = 4.68x10-40) (**S4 Table in S1 File**) and 17 of 24 genes were differentially expressed in a direction consistent with the proliferation of stem cells (70.8%, Z = 2.51, p = 2.69E-24) (**S5 Table in S1 File**).

**Table 2:** Predicted upstream regulators of 278 differentially expressed genes identified in the transcriptome of splenic myeloid cells from hamsters with VL.

Upstream Regulator (Gene symbol)	Fold change	Activation Z- score	p-value	Targets	Regulators
CSF2	6.4	3.0	4.10E-18	36	165 (18)
TLR4	2.4	2.8	2.50E-22	37	165 (20)
IFNG	11.1	2.4	3.09E-46	95	174 (19)
CALCA	3.1	2.2	3.49E-08	11	157 (18)
HRAS	7.2	2.1	1.78E-25	49	184 (24)
IL4	10.6	2.1	5.95E-22	52	157 (17)
FPR2	3.3	2.0	2.64E-05	4	148 (20)
JUNB	4.4	1.8	4.68E-14	18	183 (16)
IL6	5.0	1.9	9.23E-38	67	173 (17)

Colony stimulating factor 2 (CSF2); toll like receptor 4 (TLR4); interferon gamma (IFNG); calcitonin related polypeptide alpha (CALCA); HRas proto-oncogene (HRAS); interleukin 4 (IL4); JunB proto-oncogene (JUNB); formyl peptide receptor 2 (FPR2); interleukin 6 (IL6). Activation Z-score given by the number of gene targets in data set (Predicted with IPA).

To determine whether the myeloid cell division was associated with a dysregulated cell cycle, we analyzed a subset of proto-oncogenes and tumor suppressor genes expressed in the transcriptome. Known proto-oncogenes and tumor suppressor genes typical of cancer were identified (**Fig 4D and Table 3**). Thirteen percent (9/72) of proto-oncogenes were upregulated and 4.5% (2/44) of tumor suppressor genes were down regulated (**Table 3**). To our knowledge, this is the first demonstration of dysregulation of cell cycle control in experimental visceral leishmaniasis. Likewise, analysis of the regulatory network of proto-oncogenes and tumor suppressor genes predicted a process like proliferation of tumor cell lines (Z = 2.6) (**Fig 4E**). Interestingly, genes that we found associated with splenic myeloid cell proliferation in experimental VL, are drug targets for treatment of cancer diseases (**Table 4**).

**Table 3:** Differentially expressed proto-oncogenes and tumor suppressor genes identified in the transcriptome of splenic myeloid cells from hamsters with VL.

Gene Symbol	Fold Change	Family
**Proto-oncogenes**		
HRAS	7.2	GTPase enzyme
JUNB	4.4	AP1 transcription factor subunit
MYB	4.1	transcription regulator
FGR	3.8	Kinase (Src family tyrosine kinase)
FYN	3.8	Kinase (Src family tyrosine kinase)
SRC	3.8	Kinase (non-receptor tyrosine kinase)
CBL	2.1	transcription regulator
CBLB	2.1	Enzyme
ROS1	2.0	Kinase (receptor tyrosine kinase)
FES	-5.1	Kinase (tyrosine kinase)
FOSB	-5.2	AP-1 transcription factor subunit
BLK	-6.1	Kinase (Src family tyrosine kinase)
**Tumor suppressor genes**		
VHL	-10.6	transcription regulator
RPL10	-4.3	Other

HRas proto-oncogene (HRAS); JunB proto-oncogene, AP1 transcription factor subunit (JUNB); MYB proto-oncogene (MYB); FGR proto-oncogene (FGR); FYN proto-oncogene (FYN); SRC proto-oncogene (SRC); Cbl proto-oncogene (CBL); Cbl proto-oncogene B (CBLB); ROS proto-oncogene 1 (ROS1); FES proto-oncogene (FES); FosB proto-oncogene (FOSB); BLK proto-oncogene (BLK); von Hippel-Lindau tumor suppressor (VHL); ribosomal protein L10 (RPL10). Differentially expressed genes (FDR<0.05 and a fold change of 2.0, with reference to uninfected).

**Table 4:** Targets of drug development in cancer diseases found overexpressed in the transcriptome of splenic myeloid cells of hamsters with VL.

Gene Symbol	Fold Change	Family	Drugs
HRAS	7.2	Enzyme (GTPase)	1
IL6	5.0	Cytokine	2
CD44	4.5	Other	3
FGR	3.8	Src family tyrosine kinase	4
FYN	3.8	Src family tyrosine kinase	5
SRC	3.8	non-receptor tyrosine kinase	6
PRKCI	3.7	Kinase	7
STAT3	3.3	transcription regulator	8
IGF2	2.3	growth factor	9
ROS1	2.0	Kinase	10

HRas proto-oncogene (HRAS); interleukin 6 (IL6); CD44 molecule (Indian blood group) (CD44); FGR proto-oncogene (FGR); FYN proto-oncogene, (FYN); SRC proto-oncogene (SRC); protein kinase C iota (PRKCI); signal transducer and activator of transcription 3 (STAT3); insulin like growth factor 2 (IGF2); ROS proto-oncogene 1, receptor tyrosine kinase (ROS1).

**Fig 5:**
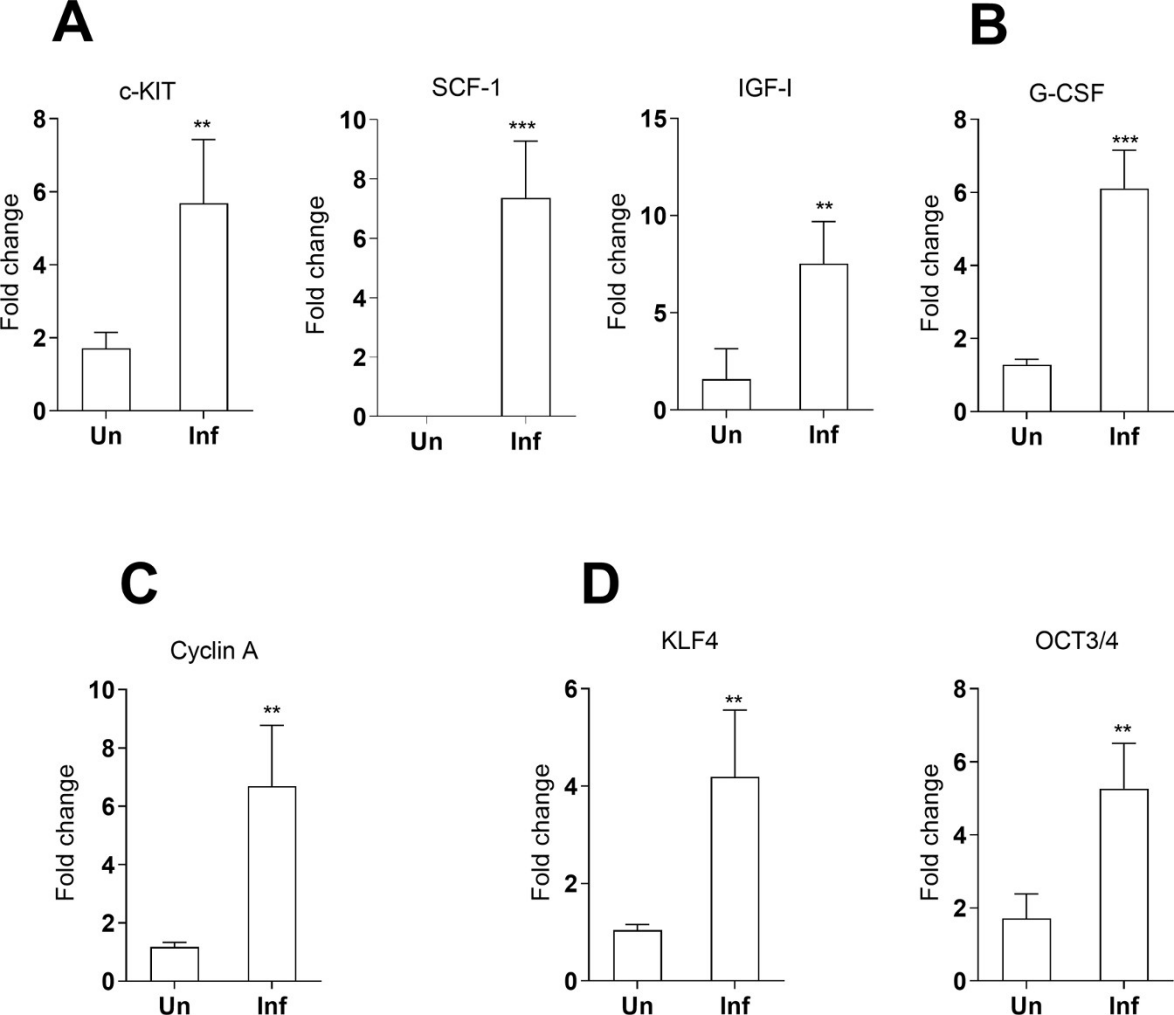
Expression of genes associated with stem cells in the spleen of hamsters with VL as determined by real time qPCR. **A-C**, Genes expressed by splenic adherent myeloid cells; **D**, Genes expressed in total spleen of uninfected hamsters (Un) or hamsters infected with *L*. *donovani* (Inf) at 28d p.i. Data represents fold change with reference to uninfected (mean and SE of 3 experiments, 8 hamsters per group per experiment). c-KIT: p = 0.03; SCF: p = 0.0067, G-CSF: p = 0.0001; IGF-1: p = 0.0051; cyclin A: p = 0.0048; KLF4: p = 0.0064; OCT3/4: p = 0.02, Mann-Whitney Test.

Selected genes upregulated in the splenic myeloid cell transcriptome associated with stemness or cell self-renewal capacity were verified by qPCR. This included stem cell factor (SCF1) and its cognate receptor tyrosine-protein kinase KIT (c-KIT) (**Fig 5A**); the growth factors G-CSF and IGF-1 (**Fig 5B**) which participate in the production of stem cells; and cyclin A (**Fig 5C**) which regulates cell entry into mitosis. Markers of pluripotency KLF4 and OCT3/4 were upregulated in total spleen tissue (**Fig 5D**) but not in purified splenic myeloid cells, suggestive of an environment supportive of self-renewal. Together these data suggest that the genes involved in the local proliferation of myeloid cells are regulated by processes akin to stem cells.

### Expansion of the splenic myeloid cell population is regulated through PI3K/AKT, MAPK and STAT3 signaling pathways

**Fig 6:**
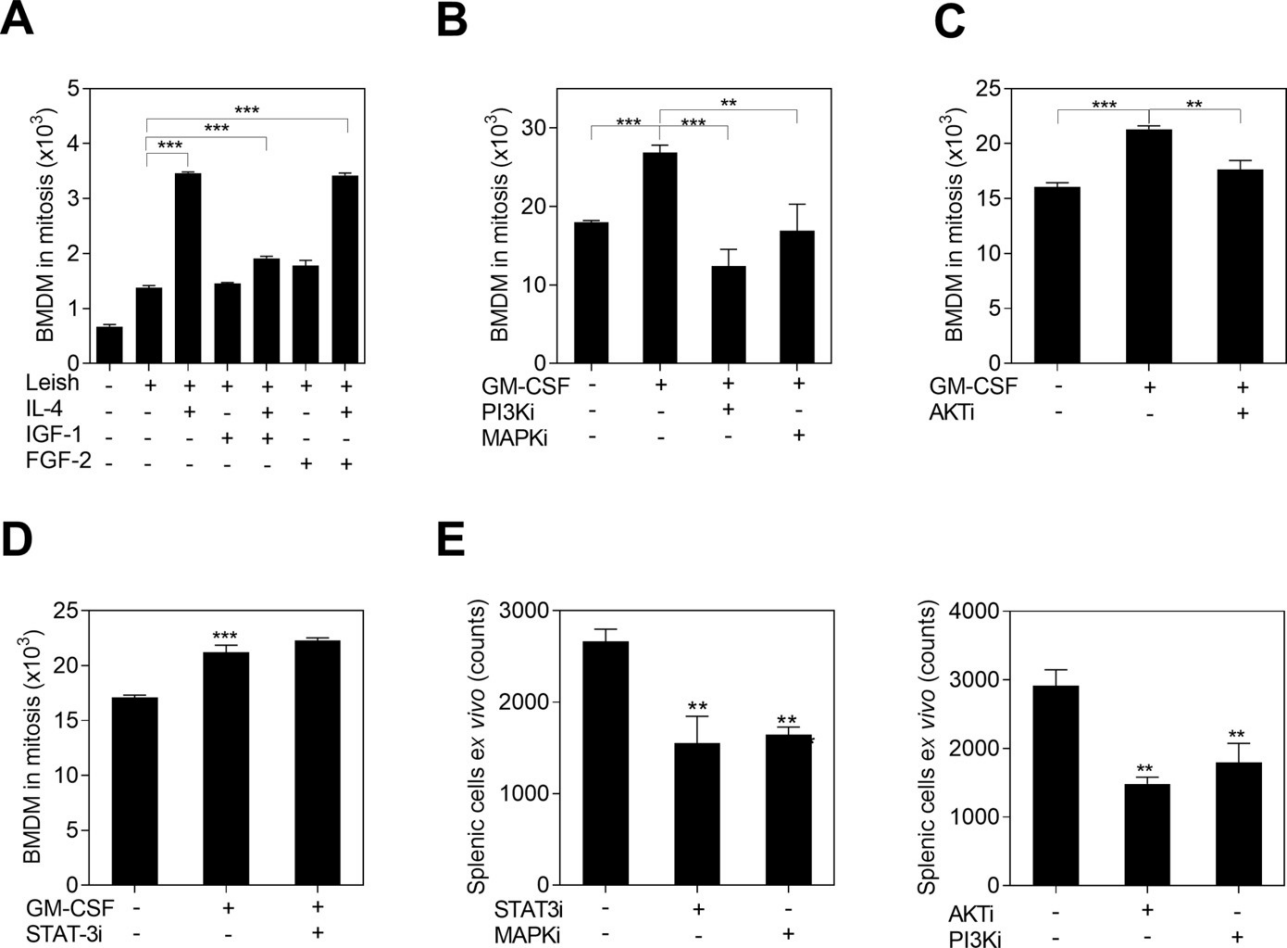
The proliferation of myeloid cells exposed to *L*. *donovani* depends on PI3K/AKT, MAPK and STAT3 signaling. **A,** Mitosis of BMDM (ki-67+) after 72h of *in vitro* exposure to *L*. *donovani* (Leish) and stimulation with IGF-1, FGF-2 or IL-4 (***p<0.001, Dunnett Multiple Comparisons Test); **B**, **C**, Mitosis of BMDM (ki-67+) after exposure to *L*. *donovani*, stimulation with GM-CSF, and treatment with either 1.5μM PI3-kinase inhibitor (LY29402), 10μM MAPK kinase inhibitor (PD98059), or 0.2uM AKT inhibitor (MK-2206) (***p<0.001, **p<0.01, Tukey-Kramer Multiple Comparisons Test); **D,** Mitosis of BMDM (ki-67+) after exposure to *L*. *donovani*, GM-CSF and 25μM STAT3 inhibitor (S31-201); Cells in mitosis = proportion of Ki-67+ cells by flow cytometry x number of cells/100; **E**, Number of splenic myeloid cells from infected hamsters after *ex vivo* treatment with the same concentrations of chemical inhibitors (***p<0.001, **p<0.01, Tukey-Kramer Multiple Comparisons Test, with reference to untreated cells).

To verify the participation of predicted regulators of cell proliferation, we overlaid the fold-change of differentially expressed genes in the transcriptome with signaling proteins downstream of receptor tyrosine kinase (RTK) and cytokine receptor signaling. We found that the phosphoinositide 3-kinase and protein Kinase B (PI3K/AKT) signaling, STAT3, signal transducer and activator of transcription 5 (STAT5), signal transducer and activator of transcription 1 (STAT1) and rat sarcoma and mitogen-activated protein kinase (RAS/MAPK) signaling converge at the functions of cell proliferation, cell differentiation and cell survival (**S2 Fig in S1 File**). To validate putative upstream regulators of proliferation, we exposed BMDM to *L*. *donovani* and growth factors (IGF-1 FGF-2 and GM-CSF) or IL-4 to activate PI3K/AKT signaling. We found significantly increased proliferation of cells exposed to IL-4 and GM-CSF compared with untreated infected cells (**Fig 6A and 6B**). IGF-1 and FGF-2 did not increase proliferation of infected cells (**Fig 6A**). We then targeted downstream signaling proteins of these pathways with chemical inhibitors to measure the effect on cell division (**S2 Fig in S1 File**). We found that PI3-kinase inhibitor (LY29402), MAPK inhibitor (PD98059) and AKT inhibitor (MK-2206) suppressed BMDM proliferation induced by *in vitro* stimulation with parasites and GM-CSF (**Fig 6B and 6C**). A similar reduction of proliferation was observed in splenic myeloid cells from infected animals exposed to those inhibitors *ex vivo* (**Fig 6E**). Although, the STAT3 inhibitor (S31-201) did not affect the proliferation of *in vitro* cultured BMDM (**Fig 6D**), it decreased proliferation of *ex vivo* cultured splenic myeloid cells isolated from infected hamsters (**Fig 6E**). This result indicated that cell proliferation mediated through STAT3 signaling was active in the *in vivo* setting. Inhibition of MAPK, AKT and PI3K similarly reduced proliferation of splenic myeloid cells in the *ex vivo* model (**Fig 6E**). We confirmed that none of the small molecule inhibitors affected cell viability or apoptosis in the *ex vivo* assay at 48h (**S3A and S3B Fig in S1 File**).

**Fig 7:**
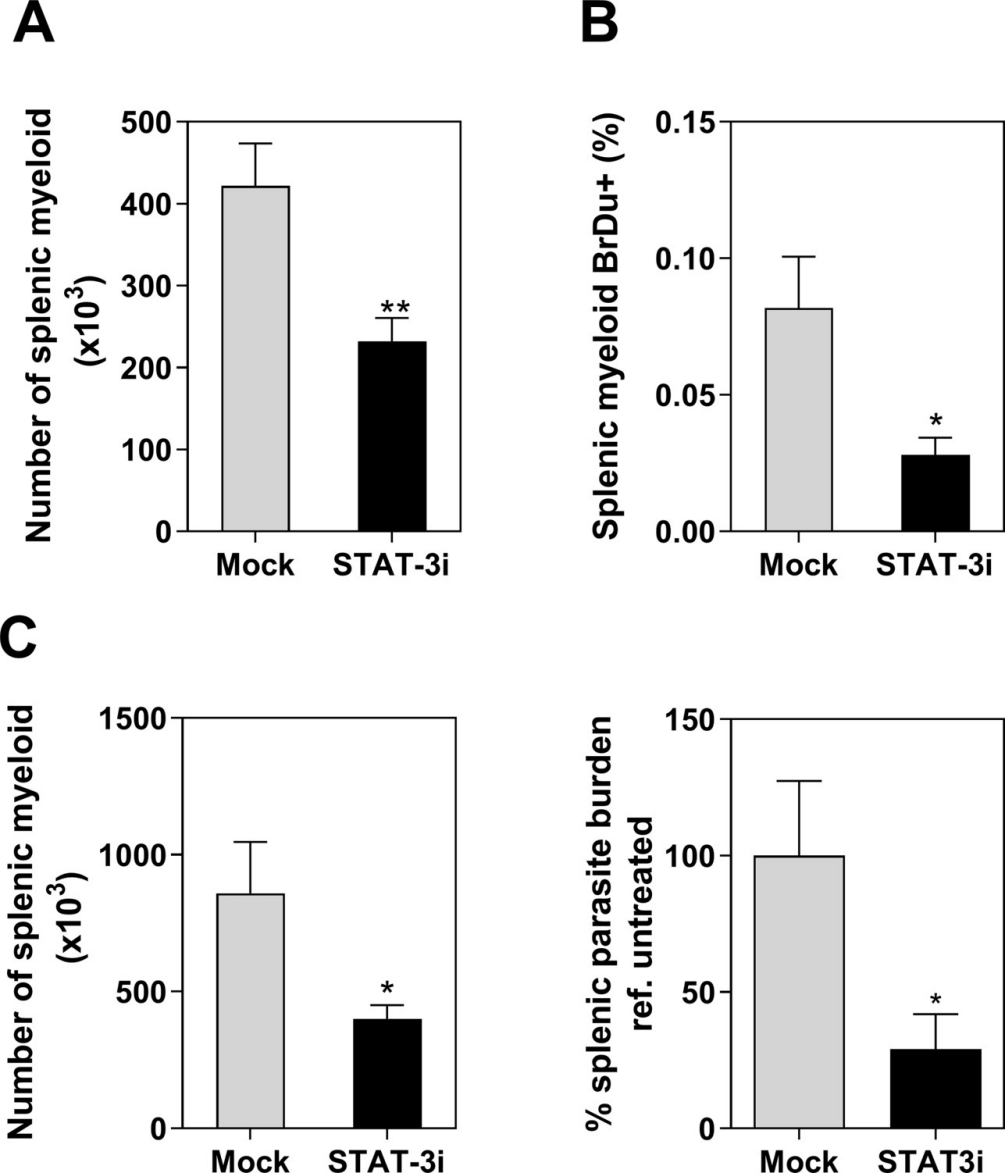
STAT3 inhibitors modulate the proliferation of splenic myeloid cells in hamsters infected with *L*. *donovani*. **A,** Number of splenic myeloid cells recovered from infected hamsters after treatment with STAT3 inhibitor (cucurbitacin) starting 14 days p.i.(light microscopy) (** p = 0.007, Unpaired t test); **B,** Proportion of in situ proliferative splenic myeloid cells from the same groups of hamsters injected i.p. with BrdU (BrdU pulse-chase incorporation, flow cytometry) (* p = 0.03, Unpaired t test); **C**, Number of myeloid cells and Percentage of splenic parasite burden in hamsters infected with *L*. *donovani* treated with the STAT3 inhibitor (Stattic) starting at 18–21 days p.i. Percentage of parasite burden in treated vs. mock treated hamsters (RT qPCR) (*p = 0.02, Mann Whitney test, n = 9–10 hamsters per group, 2 experiments).

Since proliferating myeloid cells accumulate in spleen and appear to be more permissive to *L*. *donovani* infection, we reasoned that the myeloid cell proliferation in VL could be a therapeutic target. Search for gene targets among cancer-related pathways revealed the transcription factor STAT3 as a potential target (**Table 4**). We found that STAT3 was directly or indirectly involved in the regulation of 42 of 106 genes (40%) common to proliferation and growth of tumors (**S6 Table in S1 File**). STAT3 also was involved in the regulatory network of genes leading to a pathogenic production of phagocytes and differentiation of macrophages (CSF2, CSF1, CSF2RB, IFI16, IL-10, IL2RA, IL-4, IL-6). Therefore, we treated hamsters infected with *L*. *donovani* with a STAT3 inhibitor for two weeks. At the end of the treatment (28 days post-infection) we found that the spleens of treated animals had reduced numbers of splenic myeloid cells (p = 0.007) (**Fig 7A**). The proportion of myeloid cells proliferating *in situ*, as determined by splenic BrdU pulse-chase incorporation, was also reduced (p = 0.03) (**Fig 7B**). Importantly, reduced numbers of myeloid cells in hamsters treated with a STAT3 inhibitor were associated with a marked decrease of splenic parasite burdens (p = 0.02) (**Fig 7C**). Collectively these data expand the evidence from our previous work [17] that STAT3 is a central regulator of the pathogenesis of VL.

## Discussion

This study demonstrates that *in-situ* proliferation contributes to the accumulation of myeloid cells in the spleen in progressive VL. This proliferation is induced and/or supported by factors produced by spleen cells during disease onset. Infected cells demonstrated less proliferative capacity compared with uninfected cells, but proliferative, newly generated myeloid cells were more permissive to *L*. *donovani* replication. Interrogation of the splenic myeloid cell transcriptome [17, 18] revealed a gene signature similar to proliferating tumor cells. This included dysregulated expression of proto-oncogenes and activation of PI3K/AKT, MAPK and STAT3 signaling pathways. Inhibition of STAT3 signaling reduced *in situ* proliferation, accumulation of monocyte-like myeloid cells and decreased splenic parasite loads.

We found evidence of proliferation in splenic myeloid cells by increased incorporation of BrdU, expression markers of cell mitosis, and a 10-fold increased generation of cells in a colony forming assay. Myeloid cell colony formation was augmented by exposure to factors produced by spleen cells of hamsters infected with *L*. *donovani*. This proliferation was predicted to be induced by exposure to known drivers of monocyte proliferation (IL-4, GM-CSF) [23]. *In vitro* experiments demonstrated that proliferation was prevented after intracellular infection and M2-polarization, even in presence of GM-CSF and IL-4, which are potent inducers of BMDMs proliferation.

Colony formation assays suggested that newly generated daughter cells supply a more permissive environment for *Leishmania* replication. This is consistent with the idea that the pathogenesis of *Leishmania* infection involves generation of immature safe targets that are less effective in killing parasites through cytokine-mediated activation [8]. The anti-*Leishmania* activity of these cells is likely to be different from immature MDSCs, since mouse MDSCs were demonstrated to produce nitric oxide to kill *L*. *major* infecting the skin [24]. Even though hamster splenic myeloid cells have suppressive function [25] and express both IDO and ARG1 [17] like MDSCs, spleen cells from hamsters with progressive VL produce very low levels of nitric oxide [15, 20].

Our *in vitro* studies indicated reduced proliferation of myeloid cells containing intracellular parasites compared with those cells without intracellular parasites. Since intracellular parasites affect host cell signaling and function, it is not surprising that they affect proliferation. The parasite’s energy requirements for intracellular growth and replication may lead to a deficit in cellular energy required for host cell proliferation. Furthermore, intracellular *Leishmania* scavenge host polyamines, which are products of arginine metabolism used for parasite growth [20, 26], thereby impairing that proliferation. Increased glycolysis and activation of the tricarboxylic acid cycle is necessary for host cell proliferation, but may be hijacked to provide fuel for parasite growth and replication [27]. Intracellular infection also interferes with inflammatory pathways identified in our study, which are crucial for myeloid proliferation such as the MyD88-dependent signaling pathways [28] the MAPK signaling [29] and JAK2-STAT3 signaling. Although intracellular amastigotes impaired cell proliferation, infected cells may trigger proliferation of non-infected cells through paracrine crosstalk [30]. This may be a mechanism whereby *Leishmania* promotes the availability of host cells for its own use.

Splenic myeloid cell proliferation was maximal at 14 and 21 days p.i. and decreased thereafter. The reduced proliferation evident at 28 day p.i. coincides with a dramatic increase in parasite burden and polarization of myeloid cells toward the M2 phenotype [20, 21]. *Leishmania* infection of macrophages is known to cause M2-polarization [20, 31]. Our finding that M2 BMDMs displayed reduced proliferation, and that intracellular infection also reduced proliferation, is consistent with this knowledge. In line with these results, silencing the canonical M2 regulator STAT6 in BMDM led to increased cell proliferation following exposure to *L*. *donovani*. Collectively, these observations suggest an inverse relationship between myeloid cell proliferation and M2-polarization. This conclusion seems to conflict with our observation that IL-4 induced proliferation of myeloid cells and the recognized role of IL-4 in macrophage proliferation in other models [11, 32]. In our system, IL-4 can induce proliferation of uninfected cells, especially if they have been conditioned in the environment of the infected spleen, but the effect is blocked by intracellular infection. We conclude that parasite proliferation overrides host cell proliferation.

Analysis of the splenic myeloid cell transcriptome revealed significant similarities with proliferative cancer cells. Growth factors (CSF1, G-CSF, IL-6 and SCF) and TLR4 activation identified in our study are signals that promote disease in hematopoietic malignancies [33–37]. The MAPK pathway interacts with the PI3K/AKT pathway to produce overlapping effects on proliferation and survival of tumor cells [35, 38]. IL-6 and TLRs activate STAT3, a key transcription factor that regulates genes implicated in cell proliferation, cell cycle progression, cell survival, and angiogenesis in cancer [39, 40]. We validated some of those *in-silico* predictions and found that chemical inhibitors of downstream signals reduced *in vitro* and *ex vivo* cell division. The predicted similarities of myeloproliferative cancer diseases with splenic myeloid proliferation in VL is in line with some clinical similarities in VL and myeloproliferative disorders [41].

Dysregulation of mechanisms that control the cell cycle are associated with aberrant cell proliferation in cancer disease [42]. Increased frequency of proliferative progenitors in S phase of the cell cycle was reported in spleen of BALB/c mice infected with *L*. *donovani* [7]. Accordingly, our analysis of a subset of genes that had a significant Z score for similarity to proliferation of tumor cells predicted cell cycle progression through genes such as CDK4, HRAS, IL-4, CSF2, IFNG, IL-6 and STAT3 [43]. CDK4 is known to accelerate the G1 and G2 mitotic phases, providing a cell division advantage to cancer cells [44].

Myeloid cell proliferation was predicted through upstream regulators HRAS and JUNB proto-oncogenes. In agreement with this, leukemic cells with a myelomonocytic phenotype overexpress proto-oncogenes HRAS, JUNB, CBL and FGR [43]. Proto-oncogene overexpression, in conjunction with downregulation of tumor suppressor genes (VHL, RPL10), suggest a lower stringency of cell cycle checkpoints that may cause failure of cell cycle arrest. To our knowledge, this is the first time a checkpoint failure is associated with dysregulated cell cycle and myelopoiesis in VL.

Our analysis of the splenic myeloid cell transcriptome showed a significant Z score for similarity to stem-cell proliferation. Increased expression of growth factor genes (SCF1, c-KIT, GM-CSF, IGF-2, FGF-2) and self-renewal genes (SRC, MYB, FYN, MYCN, OCT4, KLF4) suggested stem cell-like mechanisms of proliferation [45, 46]. The expansion of hematopoietic progenitors was predicted through increased gene expression and predicted activation of canonical pathways associated with that function (RAS, GM-CSF, CSF1, CCL3, AHR, ICOS, IFNG, IL-10, IL-4, IL-6, MYB, STAT3, STAT5, TLR4, OCT3/4, KLF4) [47]. This is strongly supported by previous studies showing proliferative hematopoietic myeloid progenitors amplified in the spleen of both BALB/c mice and hamsters infected with *L*. *donovani*. Altogether, these observations agree with previous studies that indicated the infected spleen provided the environment needed to support myeloid cell proliferation from hematopoietic cell precursors [7, 9, 30].

In summary, we demonstrated the *in-situ* proliferation of about 4–6% of splenic myeloid cells in a model of progressive VL. Spleen cells and the infected splenic milieu take on a genetic signature similar to that of tumor cells and the tumor environment, which leads to the generation of myeloid cells permissive to *Leishmania* infection. Myeloid cell proliferation in the M1-like inflammatory splenic environment shared transcriptional regulators of myeloproliferative cancer cells. Most notably, STAT3 reduced excessive splenic myeloid cell proliferation and parasite burden, highlighting their potential as target of host-directed therapy. Viewing the accumulation of disease-promoting myeloid cells from this perspective opens new areas of investigation that will help to understand the pathogenesis of VL.

## Supporting information

S1 File (PDF)

S1 Checklist The ARRIVE essential 10: Author checklist. (PDF)
