# Supplementary material for: In-situ proliferation contributes to the accumulation of myeloid cells in the spleen during progressive experimental visceral leishmaniasis
Source: PLoS One. 2020 Nov 12;15(11):e0242337. doi: 10.1371/journal.pone.0242337 (PMC7660562; doi:10.1371/journal.pone.0242337)
Supplement: S1 File — (PDF) [file pone.0242337.s001.pdf]

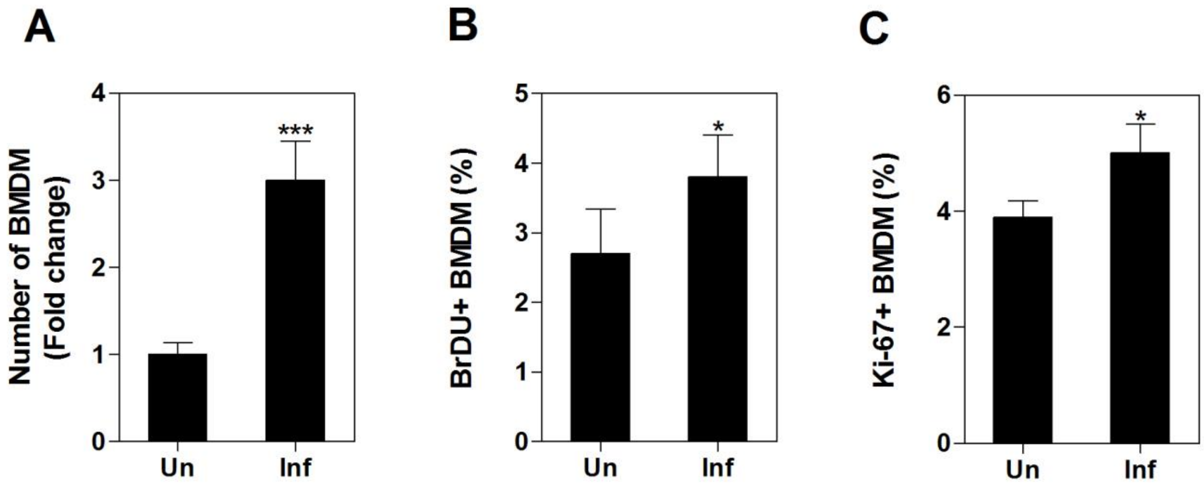
A
B
C
Number of BMDM
(Fold change)
BrDU+ BMDM (%)
Ki-67+ BMDM (%)
***
*
*
Un
Inf
0
1
2
3
4
5
6

S1 Figure.

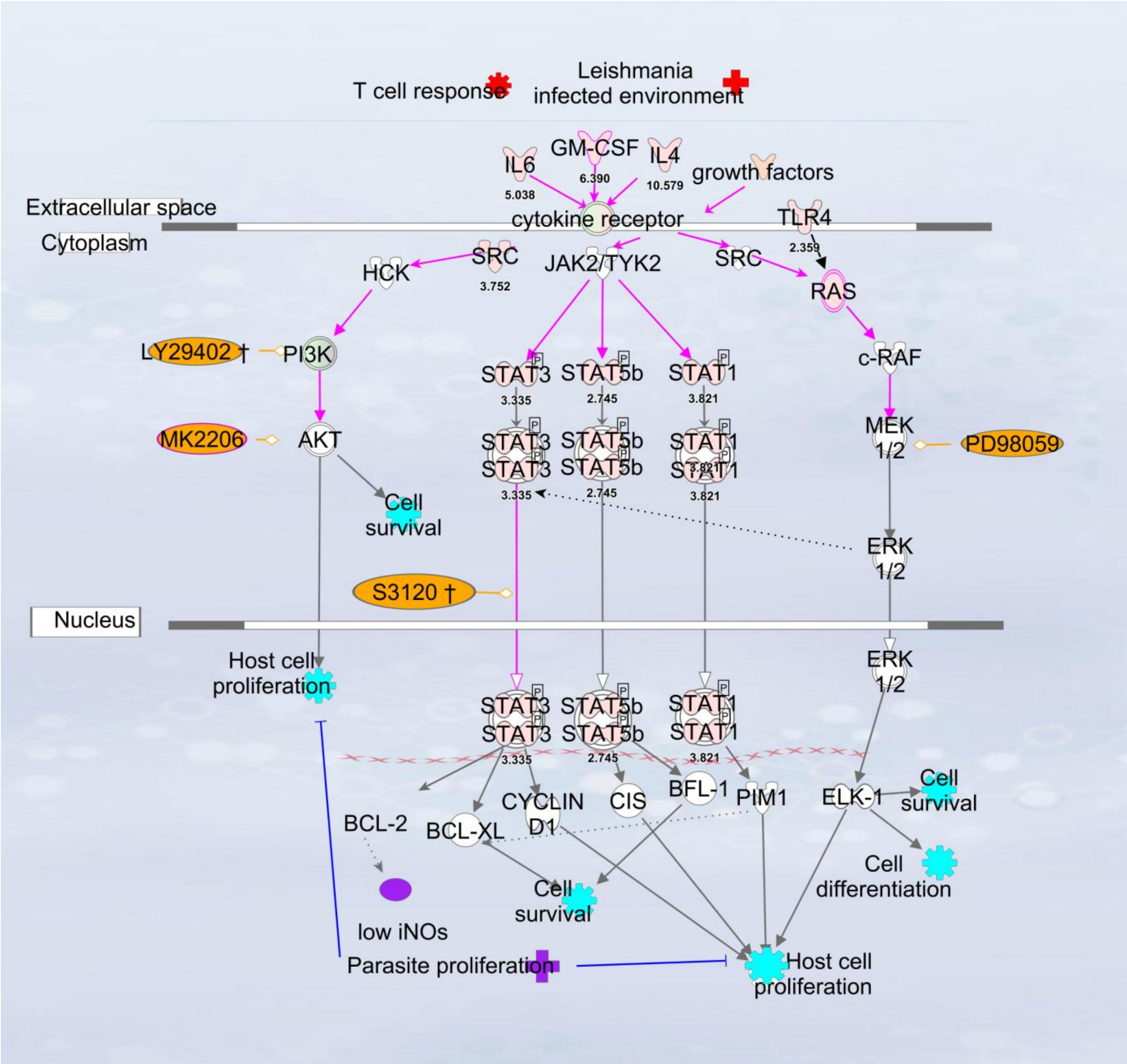
T cell response
Leishmania infected environment
IL6
5.038
GM-CSF
6.390
IL4
10.579
growth factors
Extracellular space
Cytoplasm
cytokine receptor
TLR4
2.359
SRC
3.752
HCK
JAK2/TYK2
SRC
RAS
LY29402 †
PI3K
STAT3
3.335
STAT5b
2.745
STAT1
3.821
c-RAF
MK2206
AKT
MEK 1/2
PD98059
Cell survival
ERK 1/2
S3120 †
Nucleus
Host cell proliferation
ERK 1/2
CYCLIN D1
CIS
BFL-1
PIM1
ELK-1
Cell survival
BCL-2
BCL-XL
Cell differentiation
low iNOs
Parasite proliferation
Host cell proliferation

S2 Figure.

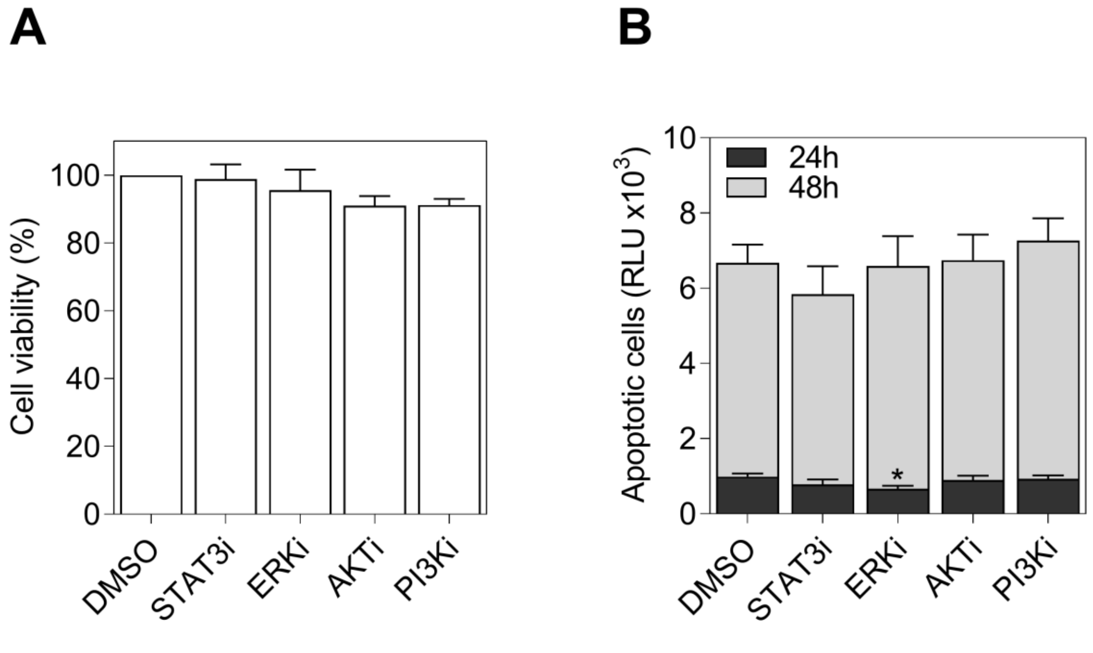
A
B
Cell viability (%)
0
20
40
60
80
100
DMSO
STAT3i
ERKi
AKTi
PI3Ki
Apoptotic cells (RLU x10^3)
0
2
4
6
8
10
24h
48h
*
DMSO
STAT3i
ERKi
AKTi
PI3Ki

S3 Figure.

S1 Table. Genes expressed by splenic myeloid cells of hamsters with visceral leishmaniasis (VL) with a measurement direction consistent with proliferation of tumoral cells (141 of 278 genes, 50%, Z=2.05, p=2.45E-27). Prediction in concordance with literature findings (IPA software).

ID	Genes in dataset	Prediction	Fold Change	Findings
AGAP2	ArfGAP with GTPase domain, ankyrin repeat and PH domain 2	Increased	223.9	Increases (3)
AHSG	alpha 2-HS glycoprotein	Increased	2.3	Increases (1)
ANXA2	annexin A2	Increased	2.3	Increases (4)
ANXA6	annexin A6	Increased	-2.3	Decreases (1)
APC	APC, WNT signaling pathway regulator	Increased	-29.8	Decreases (6)
AR	androgen receptor	Increased	2.7	Increases (83)
ARID4B	AT-rich interaction domain 4B	Increased	-2.4	Decreases (1)
ATG7	autophagy related 7	Increased	2.6	Increases (5)
ATP6V0A1	ATPase H+ transporting V0 subunit a1	Increased	4.4	Increases (1)
BDNF	brain derived neurotrophic factor	Increased	3.9	Increases (8)
CALCA	calcitonin related polypeptide alpha	Increased	3.1	Increases (2)
CASP8	caspase 8	Increased	11.4	Increases (7)
CBL	Cbl proto-oncogene	Increased	2.1	Increases (2)
CCL17	C-C motif chemokine ligand 17	Increased	4.5	Increases (1)
CCL3	C-C motif chemokine ligand 3	Increased	3.3	Increases (4)
CD28	CD28 molecule	Increased	7.8	Increases (10)
CD4	CD4 molecule	Increased	-4.0	Decreases (1)
CD44	CD44 molecule (Indian blood group)	Increased	4.5	Increases (26)
CD72	CD72 molecule	Increased	-5.0	Decreases (1)
CDK4	cyclin dependent kinase 4	Increased	2.7	Increases (9)
CEACAM1	carcinoembryonic antigen related cell adhesion molecule 1	Increased	-8.7	Decreases (8)
CIRBP	cold inducible RNA binding protein	Increased	14.9	Increases (2)
COL18A1	collagen type XVIII alpha 1 chain	Increased	-6.2	Decreases (5)
CSF1	colony stimulating factor 1	Increased	4.4	Increases (5)
CSF2	colony stimulating factor 2	Increased	6.4	Increases (20)
CTSD	cathepsin D	Increased	-6.6	Decreases (3)
CYP1A1	cytochrome P450 family 1 subfamily A member 1	Increased	17.8	Increases (5)
CYP1B1	cytochrome P450 family 1 subfamily B member 1	Increased	2313.2	Increases (1)
CYR61	cysteine rich angiogenic inducer 61	Increased	3.3	Increases (24)
DDX5	DEAD-box helicase 5	Increased	3.5	Increases (1)
DEGS1	delta 4-desaturase, sphingolipid 1	Increased	3.7	Increases (4)
DKK3	dickkopf WNT signaling pathway inhibitor 3	Increased	-5.2	Decreases (3)
DMTF1	cyclin D binding myb like transcription factor 1	Increased	4.6	Increases (1)
DNAJB4	DnaJ heat shock protein family (Hsp40) member B4	Increased	-3.4	Decreases (2)
DTX1	deltex E3 ubiquitin ligase 1	Increased	3.2	Increases (2)
E2F5	E2F transcription factor 5	Increased	2.2	Increases (1)
EDN1	endothelin 1	Increased	18.2	Increases (9)
EEF1A1	eukaryotic translation elongation factor 1 alpha 1	Increased	-21.3	Decreases (3)
EMP1	epithelial membrane protein 1	Increased	2.6	Increases (1)
ENTPD5	ectonucleoside triphosphate diphosphohydrolase 5	Increased	-4.7	Decreases (2)

EPS8	epidermal growth factor receptor pathway substrate 8	Increased	14.4	Increases (3)
EZH2	enhancer of zeste 2 polycomb repressive complex 2 subunit	Increased	3.7	Increases (26)
FAS	Fas cell surface death receptor	Increased	5.8	Increases (7)
FES	FES proto-oncogene, tyrosine kinase	Increased	-5.1	Decreases (3)
FPR2	formyl peptide receptor 2	Increased	3.3	Increases (4)
FXN	frataxin	Increased	-2.4	Decreases (1)
FYN	FYN proto-oncogene, Src family tyrosine kinase	Increased	3.8	Increases (3)
GDF15	growth differentiation factor 15	Increased	-2.0	Decreases (6)
GLS2	glutaminase 2	Increased	-6.2	Decreases (1)
GNRH1	gonadotropin releasing hormone 1	Increased	-8.2	Decreases (2)
GPER1	G protein-coupled estrogen receptor 1	Increased	2.1	Increases (13)
GPS2	G protein pathway suppressor 2	Increased	-5.4	Decreases (3)
H2AFY	H2A histone family member Y	Increased	-2.1	Decreases (2)
HEXIM1	hexamethylene bisacetamide inducible 1	Increased	-2.3	Decreases (1)
HMOX1	heme oxygenase 1	Increased	-3.2	Decreases (11)
HNF1A	HNF1 homeobox A	Increased	2.6	Increases (1)
HNRNPH2	heterogeneous nuclear ribonucleoprotein H2	Increased	3.3	Increases (1)
HRAS	HRas proto-oncogene, GTPase	Increased	7.2	Increases (24)
HSP90AA1	heat shock protein 90 alpha family class A member 1	Increased	3.2	Increases (2)
HSPB1	heat shock protein family B (small) member 1	Increased	6.9	Increases (2)
HVCN1	hydrogen voltage gated channel 1	Increased	2.5	Increases (1)
ICOS	inducible T-cell costimulator	Increased	4.8	Increases (5)
IDS	iduronate 2-sulfatase	Increased	-5.8	Decreases (2)
IGF2	insulin like growth factor 2	Increased	2.3	Increases (43)
IKBKE	inhibitor of nuclear factor kappa B kinase subunit epsilon	Increased	2.9	Increases (1)
IKBKG	inhibitor of nuclear factor kappa B kinase subunit gamma	Increased	3.6	Increases (3)
IL10	interleukin 10	Increased	3.1	Increases (7)
IL15RA	interleukin 15 receptor subunit alpha	Increased	3.1	Increases (2)
IL2RA	interleukin 2 receptor subunit alpha	Increased	2.9	Increases (3)
IL4	interleukin 4	Increased	10.6	Increases (24)
IL6	interleukin 6	Increased	5.0	Increases (100)
IL6R	interleukin 6 receptor	Increased	2.1	Increases (16)
ITGA2	integrin subunit alpha 2	Increased	2.5	Increases (2)
KCNN4	potassium calcium-activated channel subfamily N member 4	Increased	5.6	Increases (1)
KDM1A	lysine demethylase 1A	Increased	2.3	Increases (2)
KLF10	Kruppel like factor 10	Increased	-2.7	Decreases (1)
KLK7	kallikrein related peptidase 7	Increased	-241.3	Decreases (1)
MGEA5	meningioma expressed antigen 5 (hyaluronidase)	Increased	2.1	Increases (3)
MYB	MYB proto-oncogene, transcription factor	Increased	4.1	Increases (13)
MYCN	MYCN proto-oncogene, bHLH transcription factor	Increased	4.1	Increases (10)
NCAM1	neural cell adhesion molecule 1	Increased	2.7	Increases (1)
NPY	neuropeptide Y	Increased	-3.0	Decreases (1)
NTF3	neurotrophin 3	Increased	2.6	Increases (6)
PDIA3	protein disulfide isomerase family A member 3	Increased	-220.6	Decreases (4)
PDLIM2	PDZ and LIM domain 2	Increased	-2.1	Decreases (4)
PHGDH	phosphoglycerate dehydrogenase	Increased	2.4	Increases (7)

PLAC8	placenta specific 8	Increased	5.9	Increases (2)
PLAT	plasminogen activator, tissue type	Increased	5.1	Increases (3)
PLAU	plasminogen activator, urokinase	Increased	2.6	Increases (8)
PMEPA1	prostate transmembrane protein, androgen induced 1	Increased	-7.4	Decreases (6)
PPP1R12A	protein phosphatase 1 regulatory subunit 12A	Increased	2.2	Increases (2)
PPP1R1B	protein phosphatase 1 regulatory inhibitor subunit 1B	Increased	2.7	Increases (8)
PRKACA	protein kinase cAMP-activated catalytic subunit alpha	Increased	3.8	Increases (4)
PRKACB	protein kinase cAMP-activated catalytic subunit beta	Increased	3.8	Increases (1)
PRKCD	protein kinase C delta	Increased	-2.5	Decreases (14)
PRKCI	protein kinase C iota	Increased	3.7	Increases (9)
PRNP	prion protein	Increased	2.1	Increases (4)
PTBP1	polypyrimidine tract binding protein 1	Increased	4.6	Increases (10)
PTPRR	protein tyrosine phosphatase, receptor type R	Increased	2.1	Increases (1)
RASAL1	RAS protein activator like 1	Increased	-6.1	Decreases (3)
RBM38	RNA binding motif protein 38	Increased	-2.5	Decreases (6)
RBM5	RNA binding motif protein 5	Increased	3.4	Increases (1)
RFX1	regulatory factor X1	Increased	-2.5	Decreases (2)
RHCE	Rh blood group D antigen	Increased	-3.7	Decreases (2)
RPS6KA3	ribosomal protein S6 kinase A3	Increased	333.6	Increases (1)
RPS6KB1	ribosomal protein S6 kinase B1	Increased	5.1	Increases (6)
RXRA	retinoid X receptor alpha	Increased	-3.6	Decreases (14)
SAMD9L	sterile alpha motif domain containing 9 like	Increased	6.7	Increases (1)
SENP1	SUMO1/sentrin specific peptidase 1	Increased	-40.0	Decreases (1)
SERPINE1	serpin family E member 1	Increased	2.3	Increases (2)
SLC36A4	solute carrier family 36 member 4	Increased	3.3	Increases (1)
SLC7A11	solute carrier family 7 member 11	Increased	8.8	Increases (4)
SLPI	secretory leukocyte peptidase inhibitor	Increased	4.3	Increases (6)
SMARCB1	SWI/SNF related, matrix associated, actin dependent regulator of chromatin, subfamily b, member 1	Increased	-4.1	Decreases (8)
SMPD2	sphingomyelin phosphodiesterase 2	Increased	-3.6	Decreases (5)
SNIP1	Smad nuclear interacting protein 1	Increased	7.1	Increases (2)
SPP1	secreted phosphoprotein 1	Increased	5.9	Increases (54)
SRC	SRC proto-oncogene, non-receptor tyrosine kinase	Increased	3.8	Increases (28)
SRF	serum response factor	Increased	2.0	Increases (3)
STAT1	signal transducer and activator of transcription 1	Increased	3.8	Increases (5)
STAT2	signal transducer and activator of transcription 2	Increased	-5.2	Decreases (3)
STAT3	signal transducer and activator of transcription 3	Increased	3.3	Increases (62)
STAT5A	signal transducer and activator of transcription 5A	Increased	2.7	Increases (4)
STAT5B	signal transducer and activator of transcription 5B	Increased	2.7	Increases (8)
TFF2	trefoil factor 2	Increased	9.8	Increases (4)
TGFB3	transforming growth factor beta 3	Increased	2.6	Increases (3)
TIAM1	T-cell lymphoma invasion and metastasis 1	Increased	2.1	Increases (1)
TLR4	toll like receptor 4	Increased	2.4	Increases (1)
TMSB10	thymosin beta 4, X-linked	Increased	-3.6	Decreases (1)
TNF	tumor necrosis factor	Increased	-3.1	Decreases (48)
TNFSF10	TNF superfamily member 10	Increased	5.2	Increases (31)

TOX	thymocyte selection associated high mobility group box	Increased	5.0	Increases (4)
TPM1	tropomyosin 1	Increased	-2.4	Decreases (1)
TRPM2	transient receptor potential cation channel subfamily M member 2	Increased	5.0	Increases (4)
UBFD1	ubiquitin family domain containing 1	Increased	223.9	Increases (1)
USP8	ubiquitin specific peptidase 8	Increased	2.5	Increases (1)
UTRN	utrophin	Increased	-50.7	Decreases (2)
VAMP2	vesicle associated membrane protein 2	Increased	9.0	Increases (1)
VCAN	versican	Increased	2.4	Increases (6)
VHL	von Hippel-Lindau tumor suppressor	Increased	-10.6	Decreases (10)
YEATS4	YEATS domain containing 4	Increased	2.4	Increases (2)

S2 Table. Genes differentially expressed in the transcriptome of splenic monocyte/macrophages of hamsters with VL associated with predicted upstream regulators of myeloid proliferation (IL4, CSF2, TLR4). Prediction in concordance with literature findings (IPA software)*.

Symbol	*Entrez Gene Name*	*Fold Change*	*Findings*	*Prediction*
IL4 regulated genes				
ANXA2	annexin A2	2.3	Upregulates (1)	Activated
BDNF	brain derived neurotrophic factor	3.9	Upregulates (1)	Activated
CCL3	C-C motif chemokine ligand 3	3.3	Upregulates (6)	Activated
CD44	CD44 molecule (Indian blood group)	4.5	Upregulates (3)	Activated
CSF1	colony stimulating factor 1	4.4	Upregulates (2)	Activated
CSF2	colony stimulating factor 2	6.4	Upregulates (5)	Activated
DPP4	dipeptidyl peptidase 4	3.2	Upregulates (1)	Activated
FAS	Fas cell surface death receptor	5.8	Upregulates (3)	Activated
IFNG	interferon gamma	11.1	Upregulates (66)	Activated
IL10	interleukin 10	3.1	Upregulates (39)	Activated
IL17A	interleukin 17A	-15.2	Downregulates (7)	Activated
IL4	interleukin 4	10.6	Upregulates (37)	Activated
IL6	interleukin 6	5.0	Upregulates (38)	Activated
IRF8	interferon regulatory factor 8	-2.0	Downregulates (1)	Activated
ITGA1	integrin subunit alpha 1	3.3	Upregulates (1)	Activated
PLAU	plasminogen activator, urokinase	2.6	Upregulates (1)	Activated
SELE	selectin E	5.8	Upregulates (5)	Activated
SERPINE1	serpin family E member 1	2.3	Upregulates (1)	Activated
VDR	vitamin D (1,25- dihydroxyvitamin D3) receptor	45.7	Upregulates (4)	Activated
AHR	aryl hydrocarbon receptor	3.8	Regulates (2)	Affected
TLR4	toll like receptor 4	2.4	Regulates (6)	Affected
VEGFA	vascular endothelial growth factor A	-3.4	Regulates (1)	Affected
CSF2 regulated genes				
CCL3	C-C motif chemokine ligand 3	3.3	Upregulates (1)	Activated

CSF1	colony stimulating factor 1	4.4	Upregulates (5)	Activated
CSF2	colony stimulating factor 2	6.4	Upregulates (13)	Activated
EDN1	endothelin 1	18.2	Upregulates (1)	Activated
FAS	Fas cell surface death receptor	5.8	Upregulates (2)	Activated
ICOS	inducible T-cell costimulator	4.8	Upregulates (1)	Activated
IL10	interleukin 10	3.1	Upregulates (7)	Activated
IL4	interleukin 4	10.6	Upregulates (2)	Activated
IL6	interleukin 6	5.0	Upregulates (7)	Activated
ITGB5	integrin subunit beta 5	-3.9	Downregulates (5)	Activated
ODC1	ornithine decarboxylase 1	2.3	Upregulates (1)	Activated
PLAU	plasminogen activator, urokinase	2.6	Upregulates (1)	Activated
SPP1	secreted phosphoprotein 1	5.9	Upregulates (2)	Activated
TLR4	toll like receptor 4	2.4	Upregulates (6)	Activated
IFNG	interferon gamma	11.1	Regulates (5)	Affected
SOD2	superoxide dismutase 2	3.6	Regulates (1)	Affected
VDR	vitamin D (1,25- dihydroxyvitamin D3) receptor	45.7	Regulates (1)	Affected

TLR4 regulated genes

EDN1	endothelin 1	18.2	Upregulates (15)	Activated
TLR4	toll like receptor 4	2.4	Upregulates (15)	Activated
IL6	interleukin 6	5.0	Upregulates (179)	Activated
CCL3	C-C motif chemokine ligand 3	3.3	Upregulates (2)	Activated
CYR61	cysteine rich angiogenic inducer 61	3.3	Upregulates (2)	Activated
SOD2	superoxide dismutase 2	3.3	Upregulates (2)	Activated
CD44	CD44 molecule (Indian blood group)	4.4	Upregulates (3)	Activated
SELE	selectin E	5.821	Upregulates (3)	Activated
CSF2	colony stimulating factor 2	6.4	Upregulates (4)	Activated
FAS	Fas cell surface death receptor	5.8	Upregulates (4)	Activated
TNFSF10	TNF superfamily member 10	5.2	Upregulates (4)	Activated
IL10	interleukin 10	3.1	Upregulates (47)	Activated
IL4	interleukin 4	10.6	Upregulates (5)	Activated
IFNG	interferon gamma	11.1	Upregulates (9)	Activated
RXRA	retinoid X receptor alpha	-3.6	Regulates (1)	Activated
SPP1	secreted phosphoprotein 1	5.9	Regulates (1)	Affected
TPM1	tropomyosin 1 (alpha)	-2.3	Regulates (1)	Affected
PLAT	plasminogen activator, tissue type	5.1	Regulates (15)	Affected
HMOX1	heme oxygenase 1	-3.2	Regulates (2)	Affected
STAT5A	signal transducer and activator of transcription 5A	2.7	Regulates (3)	Affected
IL17A	interleukin 17A	-15.2	Regulates (4)	Affected
STAT1	signal transducer and activator of transcription 1	3.8	Regulates (5)	Affected

S3 Table. Differentially expressed genes with a measurement direction consistent with hematopoiesis of mononuclear cells found in the data set of 106 common genes expressed in cancer and VL. (28 of 45 genes, 62%, Z=2.83, p=4.71E-32). Prediction in accordance with literature findings (IPA software).

ID	***name***	***Prediction***	***Fold Change***	***Finding***
CYR61	cysteine rich angiogenic inducer 61	Increased	3.3	Increases (1)
AHR	aryl hydrocarbon receptor	Increased	3.8	Increases (26)
BDNF	brain derived neurotrophic factor	Increased	3.9	Increases (2)
CCL3	C-C motif chemokine ligand 3	Increased	3.3	Increases (2)
CSF1	colony stimulating factor 1	Increased	4.4	Increases (45)
CSF2	colony stimulating factor 2	Increased	6.4	Increases (63)
HRAS	HRas proto-oncogene, GTPase	Increased	7.2	Increases (8)
ICOS	inducible T-cell costimulator	Increased	4.8	Increases (20)
IFNG	interferon gamma	Increased	11.1	Increases (63)
IGF2	insulin like growth factor 2	Increased	2.3	Increases (1)
IGHM	immunoglobulin heavy constant mu	Increased	8.2	Increases (13)
IL10	interleukin 10	Increased	3.1	Increases (66)
IL4	interleukin 4	Increased	10.6	Increases (158)
IL6	interleukin 6	Increased	5.0	Increases (118)
IL6R	interleukin 6 receptor	Increased	2.1	Increases (1)
KLF10	Kruppel like factor 10	Increased	-2.7	Decreases (6)
LAIR1	leukocyte associated immunoglobulin like receptor 1	Increased	-2.1	Decreases (1)
MYB	MYB proto-oncogene, transcription factor	Increased	4.1	Increases (26)
MYCN	MYCN proto-oncogene, bHLH transcription factor	Increased	4.1	Increases (1)
SPP1	secreted phosphoprotein 1	Increased	5.9	Increases (2)
STAT1	signal transducer and activator of transcription 1	Increased	3.8	Increases (7)
STAT3	signal transducer and activator of transcription 3	Increased	3.3	Increases (22)
STAT5A	signal transducer and activator of transcription 5A	Increased	2.7	Increases (17)
STAT5B	signal transducer and activator of transcription 5B	Increased	2.7	Increases (8)
TLR4	toll like receptor 4	Increased	2.4	Increases (10)
TNFSF10	TNF superfamily member 10	Increased	5.2	Increases (5)
TOX	thymocyte selection associated high mobility group box	Increased	5.0	Increases (11)
TWIST1	twist family bHLH transcription factor 1	Increased	-2.4	Decreases (1)

S4 Table. Differentially expressed genes with a measurement direction consistent with cell cycle progression (32 of 59 genes, 54.2%, p=4.68x10-40, Z=2.34). Prediction in accordance with literature findings (IPA software).

ID	*Entrez Gene Name*	*Prediction*	*Fold Change*	*Finding*
AR	androgen receptor	Increased	2.7	Increases (9)
EDN1	endothelin 1	Increased	18.2	Increases (9)
CSF1	colony stimulating factor 1	Increased	4.4	Increases (7)
IGF2	insulin like growth factor 2	Increased	2.3	Increases (7)
STAT1	signal t. act. of transcription 1	Increased	3.8	Increases (7)
EZH2	enhancer of zeste 2 polycomb repressive complex 2 subunit	Increased	3.7	Increases (6)
ILK	integrin linked kinase	Increased	2.4	Increases (6)
IGFBP3	insulin like g.f. binding protein 3	Increased	5.5	Increases (5)
TOX	thymocyte selection associated high mobility group box	Increased	5.0	Increases (4)
HRAS	HRas proto-oncogene, GTPase	Increased	7.2	Increases (30)
CD44	CD44 molecule (Indian blood group)	Increased	4.5	Increases (3)
TGFB3	transforming growth factor beta 3	Increased	2.6	Increases (3)
IL4	interleukin 4	Increased	10.6	Increases (25)
CDK4	cyclin dependent kinase 4	Increased	2.7	Increases (24)
CCL3	C-C motif chemokine ligand 3	Increased	3.3	Increases (2)
CYR61	cysteine rich angiogenic inducer 61	Increased	3.3	Increases (2)
STAT5A	signal t. act. of transcription 5A	Increased	2.7	Increases (2)
STAT5B	signal t. act. of transcription 5B	Increased	2.7	Increases (2)
TGFB2	transforming growth factor beta 2	Increased	2.6	Increases (2)
CSF2	colony stimulating factor 2	Increased	6.4	Increases (14)
STAT3	signal t. act. of transcription 3	Increased	3.3	Increases (14)
IFNG	interferon gamma	Increased	11.1	Increases (13)
IL6	interleukin 6	Increased	5.0	Increases (13)
GADD45A	growth arrest and DNA damage inducible alpha	Increased	3.4	Increases (12)
ADORA3	adenosine A3 receptor	Increased	6.0	Increases (1)
ICOS	inducible T-cell costimulator	Increased	4.8	Increases (1)
SPP1	secreted phosphoprotein 1	Increased	5.9	Increases (1)
VCAN	Versican	Increased	2.4	Increases (1)
CEACAM1	carcinoembryonic antigen related cell adhesion molecule 1	Decreased	-8.7	Decreases (1)
DUSP1	dual specificity phosphatase 1	Decreased	-4.7	Decreases (1)
HMOX1	heme oxygenase 1	Decreased	-3.2	Decreases (1)
TIMP2	TIMP metallopeptidase inhibitor 2	Decreased	-4.3	Decreases (1)

S5 Table. Genes expressed by splenic myeloid cells of hamsters with VL with a measurement direction consistent with proliferation of stem cells (Z=2.51, p=2.69E-24). Prediction in accordance with literature findings (IPA software).

ID	Entrez Gene Name	Prediction	Fold Change	Findings
BDNF	brain derived neurotrophic factor	Increased	3.9	Increases (2)
CD44	CD44 molecule (Indian blood group)	Increased	4.5	Increases (1)
CSF2	colony stimulating factor 2	Increased	6.4	Increases (1)
EDN1	endothelin 1	Increased	18.2	Increases (1)
IGF2	insulin like growth factor 2	Increased	2.3	Increases (3)
IL17A	interleukin 17A	Increased	-15.2	Decreases (1)
IL4	interleukin 4	Increased	10.6	Increases (1)
IL6	interleukin 6	Increased	5.0	Increases (5)
ITGA1	integrin subunit alpha 1	Increased	3.3	Increases (1)
KDM1A	lysine demethylase 1A	Increased	2.3	Increases (4)
MYB	MYB proto-oncogene	Increased	4.1	Increases (2)
MYCN	MYCN proto-oncogene, bHLH transcription factor	Increased	4.1	Increases (5)
PLAT	plasminogen activator, tissue type	Increased	5.1	Increases (2)
SPP1	secreted phosphoprotein 1	Increased	5.9	Increases (1)
SRC	SRC proto-oncogene, non-receptor tyrosine kinase	Increased	3.8	Increases (2)
STAT3	signal transducer and activator of transcription 3	Increased	3.3	Increases (1)

S6 Table. STAT-3 regulated genes predicted to affect functions similar to proliferation of tumor cell lines, identified in the transcriptome of splenic macrophages of hamsters with VL. Determined in a subset of 106 common genes associated to cell proliferation and tumor growth.

Symbol	Entrez Gene Name	Fold change
AHR	aryl hydrocarbon receptor	3.8
AR	androgen receptor	2.7
CD44	CD44 molecule (Indian blood group)	4.5
CD274	CD274 molecule	-10.2
CEACAM1	carcinoembryonic antigen related cell adhesion molecule 1	-8.7
CSF2	colony stimulating factor 2	6.4
DNMT3B	DNA methyltransferase 3 beta	-2.3
DPP4	dipeptidyl peptidase 4	3.2
EZH2	enhancer of zeste 2 polycomb repressive complex 2 subunit	3.7
FAS	Fas cell surface death receptor	5.8
GADD45A	growth arrest and DNA damage inducible alpha	3.4

HMOX1	heme oxygenase 1	-3.2
ICOS	inducible T-cell costimulator	4.8
IFNG	interferon gamma	11.1
IGFBP1	insulin like growth factor binding protein 1	3.5
IKBKE	inhibitor of nuclear factor kappa B kinase subunit epsilon	2.9
IL4	interleukin 4	3.1
IL6	interleukin 6	-15.2
IL10	interleukin 10	10.6
IL17A	interleukin 17A	5.0
KDM1A	lysine demethylase 1A	2.3
MYB	MYB proto-oncogene, transcription factor	4.1
MYD88	myeloid differentiation primary response 88	-2.8
NOTCH3	notch 3	-4.5
NPY	neuropeptide Y	-3.0
PLAU	plasminogen activator, urokinase	2.6
PPP2CA	protein phosphatase 2 catalytic subunit alpha	7.6
PRKCB	protein kinase C beta	-2.2
PRKCD	protein kinase C delta	-2.5
PRKCE	protein kinase C epsilon	-2.5
SERPINE1	serpin family E member 1	2.3
SOD2	superoxide dismutase 2	3.3
SRC	SRC proto-oncogene, non-receptor tyrosine kinase	3.8
STAT1	signal transducer and activator of transcription 1	3.8
STAT5B	signal transducer and activator of transcription 5B	2.7
STMN1	stathmin 1	-6.2
TNF	tumor necrosis factor	-3.1
TNFSF10	TNF superfamily member 10	5.2
TWIST1	twist family bHLH transcription factor 1	-2.4
VCAN	Versican	2.4
